# Immune dysregulation in endometriosis: the T cell perspective

**DOI:** 10.3389/fimmu.2026.1712360

**Published:** 2026-04-01

**Authors:** Danielle J. Sisnett, Katherine B. Zutautas, Dan H. N. Vo, Kasthuri Ravishanker, Jaelis P. Holmes, Alexandra Wodz, Chandrakant Tayade

**Affiliations:** Department of Biomedical and Molecular Sciences, Queen’s University, Kingston, ON, Canada

**Keywords:** chemokines, chronic inflammation, cytokines, endometriosis, immune dysfunction, T cells

## Abstract

Endometriosis is a chronic inflammatory, hormone dependent disorder that affects more than 200 million women worldwide. Immune dysfunction has emerged as one of the predominant mechanisms facilitating endometriosis lesion growth and survival. In particular, T cell subsets are predominant effector immune cells within the complex endometriosis lesion microenvironment. T cell biology encompasses a highly regulated and diverse network of cellular differentiation, antigen recognition, and immune regulation, all of which play critical roles in immune homeostasis. This complexity becomes particularly relevant in endometriosis, as autologous lesions evade immune clearance within this sterile, non-pathogen-driven inflammatory milieu, highlighting a failure of immune surveillance and debris clearance. Indeed, aberrant T cell phenotypes, including skewed Th2 and regulatory subsets, promote an anti-inflammatory and tissue-remodeling environment in endometriosis. Despite advances in characterizing immune cell subsets, the mechanisms underlying T cell dysfunction and lesion persistence remain poorly defined. Here, we provide comprehensive insights into the diverse T cell subsets infiltrating endometriosis lesions and associated mechanisms that potentially contribute to endometriosis lesion establishment and subsequent survival. A systems-level understanding of T cell roles within the endocrine-immune microenvironment is essential for developing targeted immunotherapies and personalized interventions for this globally prevalent disease.

## Graphical Abstract

**Figure:**
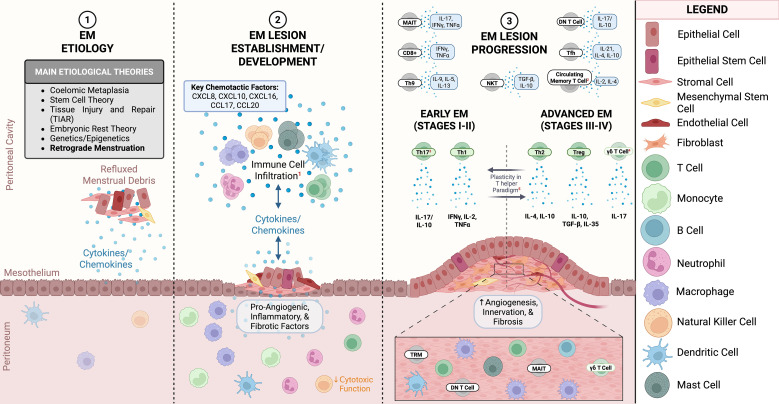
The role of T cells in endometriosis (EM) lesion development and progression. Various etiological theories exist for EM, which can co-occur, though Sampson’s theory of retrograde menstruation is most widely accepted, as visually highlighted here. This schematic depicts T cell subsets known to be dominant during early (I-II) and advanced (III-IV) stages of EM. However, as EM is a heterogenous disease, this staging system, as classified by The American Society for Reproductive Medicine (ASRM), does not always reflect the complex disease pathophysiology. T cell subsets lacking conclusive contributions to distinct EM stages are greyed out, however, this does not exclude their involvement in EM pathophysiology. Only select cytokines relevant to EM/that have been identified in EM pathophysiology have been highlighted here. Cytokine production is variable dependent on context. Graphic background adapted from a previous publication (1). ^1^While these immune cells are involved in lesion establishment/development, literature has not completely elucidated their distinct role in the initiation of lesions. ^2^Memory T cells produce a range of cytokines dependent on subset and activation status. ^3^Th17 cells are known to be either pathogenic (IL-17+) or non-pathogenic (IL-10+) in nature, however, it is not entirely clear which Th17 phenotype is involved in early versus advanced stages of EM. ^4^Data shows that γδ T cells are associated with EM disease severity, but further evidence is required to confirm this. ^5^Depicted dominance does not exclude the involvement of these subsets in other disease stages.

## 1 Introduction

Endometriosis (EM) is an estrogen-dependent gynecological disease in which endometrial-like tissue grows outside of the uterus. Impacting around 200 million reproductive-age women (assigned female sex at birth) (2) and an estimated 17–33% of transgender men (3), EM commonly presents as chronic inflammation, debilitating pelvic pain, and irregular/heavy menstruation. While exact mechanisms are not entirely known, 30–50% of patients experience infertility, with 25–50% of infertile individuals having EM (4). Due to high heterogeneity in disease presentation (5), invasive disease diagnosis, and stigmatization of reproductive disorders, patients often experience a 7–9 year diagnostic delay globally (6, 7), with nearly 75% of patients receiving a misdiagnosis during this period (8). Notably, EM symptomology substantially overlaps with several gynecological disorders, such as adenomyosis and polycystic ovary syndrome (PCOS) (9), further contributing to diagnostic delay and misdiagnosis of patients.

Despite being a leading cause of hysterectomy (10), current therapeutics are highly focused on EM symptom management and have significant side effects. While surgical excision of lesions may initially relieve symptoms, recurrence of lesions and pain symptoms is high, with recurrence rates ranging from 30–50% and 27–58% of patients undergoing re-operation within 7 years (8, 11). Patients often receive hormonal therapy as a first-line treatment to manage symptoms and/or prevent disease recurrence following surgical excision of lesions. However, this is not a long-term treatment option and cannot be used for patients aiming to conceive. The debilitating pain experienced by patients results in a significant patient, social, and economic burden, often interfering with day-to-day activities, career success, workplace productivity, mental health, and overall quality of life. Indeed, EM patients experience significantly higher health care costs compared to controls (12), with annual overall direct medical cost of EM per patient ranging from $1,459–20,239 USD in 2022 (13), and the annual economic burden of EM in the USA estimated at $69.4 billion USD in 2009 (14, 15). Given the substantial disease burden and lack of viable, effective therapeutic options, there is an urgent, unmet need to develop novel diagnostic and therapeutic targets that preserve patient fertility, reduce disease recurrence, and improve quality of life.

Though the etiology and pathogenesis of EM remain largely unknown, immune dysfunction is well-established to play a key role in the establishment and survival of lesions. Namely, altered proportions of T cell subsets, such as T regulatory (Treg) and T helper (Th)17 cells (16), have been observed systemically (peripheral blood; PB) and locally (eutopic/ectopic tissues, peritoneal fluid; PF) in EM, and are thought to drive associated chronic inflammation and immune dysregulation. Immune dysfunction, particularly changes in Treg and Th subsets, are also speculated to largely contribute to EM-associated infertility (17, 18). However, despite their clinical relevance, the contributions of specific T cell subsets to EM-associated infertility, pain, and fibrosis remain poorly characterized, representing an important gap in literature. Immune phenotypes greatly vary in EM dependent on compartment, disease stage, hormonal contraception/therapeutics, and menstrual cycle, underscoring the need to stratify results to improve comparability across studies. As EM is a hormone-dependent disease, hormonal signaling, as well as EM-associated menstrual cycle alterations, can influence T cell function and phenotype. However, hormone-mediated effects were not explored in depth as this was beyond the scope of this review. Here, we provide a comprehensive overview of key alterations in T cell subsets across compartments in EM (**Table 1**).

**Table 1:** Alterations in T cell subsets across compartments in EM.

Compartment	Cell subtype	Suggested alteration	Markers	Functional phenotype	Consensus/Key uncertainties	Analysis method	Reference(s)
Peritoneal Fluid	CD4+ T cells (CD69+)	↑ Frequency	CD4+CD8-CD69+	Activated Thelper cells	Enriched activatedCD4+ T cell phenotypein EM patientscompared to controls	CyTOFphenotyping	Guo et al., 2020 (19)
	Th1 cells	↑ *or* **–** Frequency**†** ↑ Function in early-stage EM**‡**↓ Function overall**‡**	CD4+IFNγ+ (↑)**†**; CD4+T-bet+ (**–**) N/AN/A	Type 1inflammatory signaling Elevated Th1 effector cytokine(s) Reduced Th1 effector cytokine(s)	Increased abundancein mouse model of EM(injection model)compared to controls(PBS); No changebetween EM patientsand controls TNFα elevated in early-stage EM patients,declining with severity Reduced IL-2 and IFNγin peritonealCD3+CD4+ T cellsfrom EM patients,suggesting functionalskewing	Flow cytometry Cytokine analysis (ELISA) Flow cytometry	Yuan et al., 2017**†**;Hudson et al., 2020 Pizzo et al., 2003 Mier-Cabrera et al., 2011 (20–23)
	Th2 cells	↑ *or* **–** Frequency ↑ Frequency *and* function**‡**	CD4+GATA3+ (↑); CD4+CCR4+CCR5-CCR6-CXCR3-CD25- (**–**) CD4+CCR4+CCR5-CCR6-CXCR3-CD25-CD69+	Type 2 inflammatory signaling Activated Th2 cells; Elevated Th2 effector cytokine(s)	Conflicting reports on abundance in patients compared to controls Enriched activated Th2phenotype in EMpatients compared tocontrols; supported byevidence of increasedlocal type 2 cytokines(IL-4, IL-10, IL-33) inpatients compared tocontrols	Flow cytometry;CyTOFphenotyping CyTOF phenotyping;Flow cytometry;Cytokineanalysis(multipleximmunoassay/ELISA)	Hudson et al., 2020; Guo et al., 2020;Podgaec et al., 2007; Olkowska-Truchanowicz et al., 2021; Santulli et al., 2001 (19, 22, 24–26)
	Th17 cells	↑ *or* **–** Frequency ↑ Frequency ↑ Activation/ function**‡** ↑ Function**‡**	CD4+IL-17+ (↑); CD4+CCR6+IL-17A+RORγt+ (↑); CD4+IL-17A+ (–) CD3+CD4+IL-17A+ CD3+CD4+CXCR3-CCR4+CCR10-CCR6+IL-17A +RORγt+ N/A	Pro-inflammatory(Type 17)signaling Localized Th17effectorenrichment Activated,metabolicallyreprogrammedTh17 effectorstate (IL-17-dominant) IL-17-dominant Th17 effector activity	Majority of reportssuggest increased localabundance in EMcompared to controlsand frequency iscorrelated with EMseverity Increased localabundance compared tomatched PB samples;further indications ofabundance correlatedwith EM severity Increasedactivation/metabolicreprogramming, genesignatures are stage-dependent Increased mRNAexpression of *IL17A*,*IL23A*, *RORC* in PFcells of EM patientscompared to controls;increased IL-17 proteinin EM patient PF andFF compared tocontrols; IL-17increased in EM stageI–II compared to stageIII-IV and controls, IL-17 levels wereassociated with patientinfertility	Flow cytometry Flow cytometry RNA sequencing PCR; ELISA	Pashizeh et al., 2020;Kang et al., 2023;Khan et al., 2019 Gogacz et al., 2016 Jiang et al., 2022 Khang et al., 2023;Li et al., 2024;Zhang et al., 2005 (27–33)
	Treg cells	↑ Frequency *and* – Frequency (activated Tregs) ↑ Function**‡**	CD4+FoxP3+ (↑); CD25high FoxP3+(↑); CD25+FoxP3+ (↑); CD4+FoxP3+ (↑); CD45RA-FoxP3high(–) N/A	Localized Tregenrichment associatedwith disease severity(without increasedactivation) Elevated localimmunosuppressive/anti-inflammatoryeffector cytokine(s)	Increased local abundancein EM patients compared tocontrols, which waspositively associated withEM stage/severity; however,abundance of activatedTregs was unaltered Elevated Treg effectorcytokine, sFGL2,locally in EM patientscompared to controls,which was positivelyassociated with EMstage/severity	Flow cytometry Cytokineanalysis (ELISA)	Hudson et al., 2020; Olkowska et al., 2013;Khan et al., 2019; Li et al., 2014;Tanaka et al., 2017 Hou et al., 2021(22, 30, 34, 35)
	CD8+ T cells	↓ Frequency *but* ↑ Function**‡**	N/A N/A	Reduced frequency butincreasedcytotoxic/CD8+effectorcytokine(s)	Reduced abundance inEM patients comparedto controls; howeverCD8+ effectorcytokine, IFNγ, waselevated locally in EMpatients compared tocontrols	Flow cytometry	Iwasaki et al., 1993; Podgaec et al., 2007 (24, 36)
	Th9 cells	↑ Frequency	CD4+IL-9+	Elevated IL-9-associatedinflammatory helperphenotype	Increased abundance inEM patients (OMA)compared to controls;however, reports on IL-9 levels in PF areinconsistent (↑ *or* –)	Flow cytometry	Tarumi et al., 2021;Lessey et al., 2021 (37, 38)
	NKT cells	↓ Frequency	N/A	Reduced innate-like immunesurveillance	Decreased abundancein EM patientscompared to controls	Flow cytometry	Guo et al., 2012 (39)
	MAIT cells	↑ Frequency	CD3+CD161+ Vα7.2+	Enriched, activatedinnate-like T cells	Increased abundance inEM patients comparedto controls, specificallyCD4+ and CD8+ MAITpopulations wereenriched and highlyactivated in patients	Flow cytometry	Li et al., 2019 (40)
Circulation (PB/Serum/Plasma/PBMCs)	Th1 cells	↑ *or* **–** Frequency ↑ Function in early-stage EM**‡**	CD4+IFNγ+ N/A	Type 1inflammatorysignaling Altered Th1effectorcytokine(s)	Conflicting reports onabundance in patientPB compared tocontrols TNFα elevated in PB ofearly-stage EMpatients, declining withseverity	Flow cytometry Cytokine analysis(ELISA)	Takamura et al., 2015; Pashizeh et al., 2020 Pizzo et al., 2003 (21, 28, 41)
	Th2 cells	↑ *or* **–** Frequency ↑ Function**‡**	CD4+CCR4+ CXCR5-CXCR3- (↑); CD4+IL-4+ (**–**) N/A	Type 2inflammatorysignaling Elevated Th2effectorcytokine(s)	Conflicting reports onabundance in patientPB compared tocontrols Elevated type 2cytokines (IL-4, IL-10,IL-33) in patient PBand serum compared tocontrols	Flow cytometry Cytokine analysis(ELISA);Flow cytometry	Lin et al., 2024;Takamura et al., 2015 Xia et al., 2023Podgaec et al., 2010; Santulli et al., 2001 (26, 41–43)
	Th17 cells	↑ *or* – Frequency ↑ Frequency**†** ↑Function/ pathogenicphenotype**‡**	CD4+CD25-RORγt+(↑); CD3+CD4+IL-17+(↑); CD4+IL-17A+ (–) CD4+CD25-RORγt+ N/A	Pro-inflammatory(Type 17)signaling;systemic immunedysregulation Pro-inflammatory(Type 17) signaling;systemic immunedysregulation Elevated pro-inflammatoryTh17 mediators	Conflicting reports onabundance in patientPB compared tocontrols; reducedTreg: Th17 ratio Prolonged, increasedabundance in PB post-EM induction ascompared to pre-inoculation (babooninjection model);reduced Treg: Th17ratio Elevated effector andregulatory cytokines (IL-17and IL-23) EM plasmacompared to controls,suggestive of pathogenic,pro-inflammatoryTh17 phenotype	Flow cytometry Flow cytometry Cytokineanalysis(immunoassay, ELISA)	Le et al., 2021;Delbandi et al., 2025; Khan et al., 2019;Pashizeh et al., 2020 Le et al., 2022**†** Ahn et al., 2015; Sisnett et al., 2024 (28, 30, 44–47)
	Tfh cells	↑ Frequency	CD4+CXCR5+	Enhanced humoral immune activity	Increased abundance inEM patient PBcompared to controls,may reflect chronicantigenic stimulation	Flow cytometry	Lin et al., 2024 (42)
	Treg cells	↓ *or* – Frequency ↓ Frequency**†** (iTregs & nTregs)	CD25high FoxP3+(↓); CD25+FoxP3+ (–); CD4+CD25+CD127-/low (–); CD45RA-FoxP3high(activated Tregs; –) CD4+CD25-FoxP3+ (iTregs; ↓); CD4+CD25+FoxP3+ (nTregs; ↓)	Reduced/unchangedsystemicimmunotolerantphenotype Reduced systemic regulatory capacity	Conflicting reports onsystemic abundance inpatient PB compared tocontrols; abundance ofactivated Tregs wasunaltered Reduced iTregabundance in patientPB compared tocontrols as well as areduction in Treg: Th17ratio, suggestive ofsystemic inflammation;supported by reducedabundance of iTregsand nTregs in PB post-induction of EMcompared to pre-inoculation using ababoon model ofEM (laparoscopicautologous inoculationmodel)	Flow cytometry Flow cytometry	Olkowska et al., 2013; Khan et al., 2019; Lin et al., 2024;Tanaka et al., 2017 Le et al., 2021; Le et al., 2022**†** (30, 34, 42, 78)
	CD8+ T cells	↓ *or* – Frequency	Not stated (↓); CD3+CD4-CD8+ (–)	Reduced/unchangedsystemic cytotoxicphenotype	Conflicting reportson systemic abundancein patient PBcompared to controls	Flow cytometry	Iwasaki et al., 1993; Lin et al., 2024 (36, 42)
	Th9 cells	↑ Function**‡**	N/A	Elevated Th9 effector cytokine(s)	Elevated IL-9 inEM patient plasmacompared to controls	Flow cytometry	Lessey et al., 2012 (37)
	γδ T cells (Vδ1+ & (Vδ2+)	↓ Frequency (Vδ1+) *but* ↑ Function**‡** (Vδ1+) ↓ *or* – Frequency (Vδ2+) *and* ↓ Function**‡** (Vδ2+)	CD3+γδ+TCR Vδ1+ CD3+γδ+TCR Vδ2+	Altered γδVδ1+ T cellactivation/exhaustionmarker profile Altered γδ Vδ2+T cell activation/exhaustion marker profile	Decreased abundanceof Vδ1+ T cells in EMpatient PB compared tocontrols; Vδ1+ T cellsalso exhibit heightenedactivation and possiblesigns of exhaustion(elevated PD-1); Vδ1+T were also positivelycorrelated with EMseverity and painsymptoms Conflicting reports onabundance of Vδ2+ Tcells in EM patient PBcompared to controls;Vδ2+ T cells exhibitedreduced activation andcytotoxic capabilities(elevated PD-1); Vδ2+T cells were negativelycorrelated with EMseverity and painsymptoms	Flow cytometry Flow cytometry	Hudecek et al., 2021; Lin et al., 2024Lin et al., 2024Hudecek et al., 2021 (42, 48)
	NKT cells	↓ Frequency	N/A; CD14-CD19-CD3+Vα24+Vβ11+(iNKT), CD4-CD8-CD14-CD19-CD3+Vα24+Vβ11+(DN NKT)	Reduced innate-likeimmune surveillance	Decreased NKT cellabundance in EMpatient PB comparedto controls; iNKT and DN NKT cellsubsets were alsodecreased in EM patientPB compared tocontrols, particularlyduring secretory phase	Flow cytometry	Guo et al., 2012;Correa et al., 2022(19, 49)
	MAIT cells	↑ Frequency	CD3+CD161+Vα7.2+	Enriched, activatedinnate-like T cells	Increased abundance inEM patient PBcompared to controls,specifically CD4+ andCD8+ MAITpopulations wereenriched and highlyactivated in patients	Flow cytometry	Li et al., 2019 (40)
Tissues (ectopic/eutopic endometrium)	CD4+ T cells	↑ Gene signatures (eutopic) ↑ Abundance	Gene signaturedefined byImmuCellAICD4+	Adaptive immuneenrichment/T helpercell-associated activation Lesion-associatedenrichment of CD4+T cells	Inferred increasedabundance withineutopic endometriumof patientscompared to controls Increased CD4+ T cell infiltration	GSEA/ Transcriptomicdeconvolution IHC	Wu et al., 2021 Kang et al., 2023 (27, 50)
	Th1 cells	↑ *or* ↓ Genesignatures(eutopic) ↓ Frequency (ectopic -OMA)	Gene signaturedefined byxCell andImmuCellAI CD4+IFNγ+	HeterogeneousTh1-associatedsignatures Reduced local Type 1inflammatorysignaling	Conflicting reports ongene signatures ineutopic endometrium ofpatients compared tocontrols Decreased abundance inEM patient lesionscompared to controlendometrium	GSEA/ Transcriptomicdeconvolution Flow cytometry	Poli-Neto et al., 2021; Wu et al., 2021 Takamura et al., 2015 (41, 50, 51)
	Th2 cells	↓ Genesignatures(eutopic) ↑ Function**‡**(ectopic –DIE, OMA,SPE) *and* **–** Frequency (ectopic - OMA)	Gene signaturedefined by ImmuCellAIand xCell N/A CD4+IL-4+	Reduced local Type 2inflammatorysignaling Enhanced Th2-associatedeffector signalingwithout increasedTh2 abundance	Inferred decreasedabundance withineutopic endometrium ofpatients compared tocontrols Elevated Th2 cytokines(IL-4, IL-10, TSLP, IL-33)in EM patientlesions comparedto control endometrium;however, no changein local Th2 frequency	GSEA/ Transcriptomicdeconvolution PCR; IHC;Cytokine analysis(multiplex immunoassay);Flow cytometry	Poli-Neto et al., 2021; Wu et al., 2021 Antsiferova et al., 2005;Gueuvoghlanian-Silva et al., 2018; Sadat Sandoghsaz et al., 2024; Aleksieva et al., 2025; Miller et al., 2017; Takamura et al., 2015 (41, 50–56)
	Th17 cells	↑ Frequency (ectopic – OMA, SPE &eutopic)	CD4+IL-17A+	Pro-inflammatory(Type 17)signaling	Increased infiltrationinto EM patient lesionscompared to controlendometrium; increasedIL-17 in ectopic andeutopic tissues ofpatients compared tocontrols; IL-23/Th17axis genes elevated inboth ectopic andeutopic patient tissues;linked to angiogenesisand lesion persistence	Flowcytometry;IHC; Cytokineanalysis (multipleximmunoassay);PCR	Takamura et al., 2015; Kang et al., 2023; Delbandi et al., 2025; Ahn et al., 2015;Sisnett et al., 2024 (27, 41, 44–46)
	Tfh cells	↓ Frequency (ectopic) *and* ↑ Gene signatures (eutopic)	Reference signaturedefined byCIBERSORT; Gene signaturedefined by xCell	Reduced humoralimmunity inlesions/altered Tfhcellcompartmentalization	Decreased abundancewithin ectopic tissues ofEM patients comparedto matched eutopictissues; however,increased abundancewas suggested inpatient eutopicendometrium compared tocontrols	GSEA/ Transcriptomicdeconvolution	Xie et al., 2022;Wu et al., 2021 (50, 57)
	Treg cells	↓ Frequency (activatedTregs; eutopic &ectopic – OMA) ↑ Function**‡** (ectopic – OMA, SPE)	CD45RA-FoxP3high N/A	Reduced local Tregactivation Enhanced localTreg-mediatedimmunosuppressivesignaling	Decreased abundance inboth ectopic andeutopic patient tissuescompared to controls Upregulated expression ofgenes expressed byhigh-activated Tregs inectopic lesionscompared to controls,particularly interactionsbetween *CD86* and*CTLA4* wereupregulated in EM	Flow cytometry Single cellRNAsequencing	Tanaka et al., 2017 Tan et al., 2022 (35, 58)
	CD8+ T cells	↑ *or* ↓ Frequency (ectopic– OMA, SPE) *and* ↑ Gene signatures (eutopic)	Reference signaturedefined byCIBERSORT (↑); *CD8A/B* (↓)*;* CD3+CD8+ (↑); CD8+ (↑); Gene signaturedefined by xCell	Enhanced localcytotoxicsignaling	A majority of reportssuggest increasedabundance in ectopicEM lesions comparedto matched eutopicand control endometrium;increased abundanceis also suggested withinpatient eutopicendometrium ascompared to controls	GSEA/ Transcriptomicdeconvolution;single cell RNAsequencing;IHC	Zhong et al., 2021; Huang et al., 2024;Ganewatta et al., 2018; Witz et al., 1994; Wu et al., 2021 (50, 59–62)
	MemoryT cells(TEM/TCM)	↑ GeneSignatures (CD8+TEM,eutopic) *but* ↓ Gene signatures (TEM & TCM, eutopic)	Gene signaturedefined by xCell Estimated byCIBERSORT, MCP-counter, andImmuCellAIalgorithms	Altered local memory T cell distribution	Increased enrichment ofCD8+ TEM in eutopicendometrium of EMpatients compared tocontrols; however,others report decreasedTEM and TCM geneenrichment scores ineutopic endometrium ofpatients compared tocontrols, though, thisstudy did not delineateCD4/CD8 subsets	GSEA/Transcriptomicdeconvolution	Poli-Neto et al., 2021; Bunis et al., 2022; Wu et al., 2021 (50, 51, 63)
	NKT cells	↑ Gene signatures (eutopic)	Gene signaturedefined by xCell	Innate-likeimmuneenrichment	Increased enrichment ineutopic endometrium ofpatients, mostpredominately in stageI–II, compared tocontrol endometrium	GSEA/Transcriptomicdeconvolution	Poli-Neto et al., 2020 (64)
Menstrual Effluent	Th17 cells	↓ Frequency	CD45+CD3+CD4+ CCR6+IL-17A+	Altered Th17 compartmentalization	Decreased abundancein EM patientscompared to controls,which may suggestredistribution of Th17cells outside of ME	Flow cytometry	Miller et al., 2022 (65)
	CD8+ T cells	↓ Frequency(Perforin+)/ Function**‡**	CD3+CD8+ Perforin+/high	Reduced local cytotoxicity	Reduced frequency ofperforin-positive CD8+T cells in EM patientscompared to controls,which may suggest reducedcytotoxic potential	Flow cytometry	Schmitz et al., 2021 (66)
Gut (intestinal immune and microbiome compartments)	Th17 cells	↑ Frequency (laminapropria)**†**↑ Correlation with mucosal microbiome**†**	CD4+IL-17A+CD4+CD25-RORγt+	Pro-inflammatoryTh17enrichmentTh17-linked GIand urinarymicrobiomedysbiosis	Increased abundancewithin lamina propria(large intestine) in EMmouse model (injection)compared to controls EM induction(laparoscopic autologousinoculation model) inbaboons increased GIbacteria, which wascorrelated with peripheralTh17 cells at 3moand 6mo post-inoculation,urinary bacteriawas also positivelycorrelated with Th17 levels	Flow cytometry 16S ribosomalRNA genesequencing &flow cytometry	Li et al., 2024**†** Le et al., 2022**†** (33, 47)
	Treg cells	↑ Correlationwithmucosalmicrobiome**†**	CD4+CD25-FoxP3+ (iTregs); CD4+CD25+FoxP3+ (nTregs)	Treg-linked GIand urinar microbiomedysbiosis	GI microbial alpha-diversity was positivelycorrelated withcirculating nTregs(3mo) and iTregs (9mo)post-inoculation of EMas compared to pre-inoculation using ababoon EM model(laparoscopicautologous inoculationmodel), urinary bacteriawas also correlated withperipheral nTregs post-inoculation (3moand 15mo)	16S ribosomalRNA genesequencing &flow cytometry	Le et al., 2022**†** (47)

DIE, deep infiltrating endometriosis; DN, double negative; ELISA, enzyme-linked immunosorbent assay; EM, endometriosis; FF, follicular fluid; GI, gastrointestinal; GSEA, gene set enrichment analysis; IFN, interferon; IHC, immunohistochemistry; iNKT, invariant natural killer T cells; iTreg, inducible T-regulatory cells; MAIT, mucosal-associated invariant T cells; ME, menstrual effluent; N/A, not applicable/available; nTreg, natural T-regulatory cells; OMA, ovarian endometrioma lesions; PB, peripheral blood; PCR, polymerase chain reaction; PF, peripheral fluid; sFGL2, soluble fibrinogen-like protein 2; SPE, superficial peritoneal endometriosis; TEM, effector memory T cells; TNF, tumor necrosis factor; TSLP, thymic stromal lymphopoietin.

† Animal model.

‡ Inferred; requires mechanistic study confirmation.

– No change.

↓↑ Increased/decreased.

## 2 Insights into T cells in endometriosis

It is well-known that T cells are crucial regulators of the immune response and play a key role in maintaining immune homeostasis. Imbalance in T cell subsets and their functions are often associated with a variety of health complications such as autoimmune disease, chronic inflammation, and infection. As there are numerous subsets of T cells, each with distinct functions, it is crucial to obtain an understanding of how each of these populations individually and collectively play a role in health and disease. It is also important to note that around 95% of women with EM report at least 1 comorbidity, such as inflammatory bowel disease (IBD), irritable bowel syndrome (IBS), asthma, and rheumatoid arthritis (RA) (8). As several of these associated comorbidities are driven by T cell dysfunction (67–70), it is highly likely that perturbed T cell functions overlap between EM and associated comorbidities.

### 2.1 CD4+ T helper cells in endometriosis

CD4+ T cells, also known as effector/T helper (Th) cells, are termed due to their “helper function”, as they are responsible for activating other immune cells, including B cells and CD8+ T cells, to orchestrate a broader immune response. Dependent on their environment, naïve CD4+ T cells can be activated and differentiated into various subsets, such as Th1, Th2, Th9, Th17, T follicular helper cells (Tfh), and Tregs, each with distinct functions. CD4+ T cells have been increasingly examined in the context of EM due to their central role in regulating immune responses, including those promoting inflammation, a hallmark feature of EM. Literature depicts an increased percentage of CD4+ T cells in EM patient PB as compared to the CD8+ T cell population, with CD4+ T cells comprising 67% of total T cells (71). Though, proportions of T cell subsets appear to be distinct to sample type, as within patient PF CD4+ T cells only comprised 37% of total T cells (71). Within patient tissues, others report increased CD4+ T cell gene signatures within the eutopic endometrium of EM patients as compared to healthy controls (50), as well as increased infiltration of CD4+ T cells into ectopic lesions compared to controls (27). While these local and systemic alterations have been reported in literature, a systematic review of CD4+ T cells in EM found no clear correlation between EM and systemic deviations of CD4+ T cells (72).

#### 2.1.1 T helper 1 cells

Th1 cells play a critical role in eliciting a pro-inflammatory type 1 immune response, primarily focused on protecting hosts against intracellular pathogens. Th1 cells produce distinct cytokines, including IL-2, TNFα, and pro-inflammatory IFNγ, which help to promote T cell growth and differentiation as well as activate M1 macrophages and cytotoxic T cells to eliminate foreign cells/debris/pathogens. While Th1 cells do aid in host defense, overreactive Th1 responses can induce various inflammatory and organ-specific autoimmune diseases, such as RA, multiple sclerosis (MS), and type 1 diabetes (42). It has long been debated whether EM is an autoimmune disease (73), with some reporting increased Th1 cells in patient PB (41) and within the PF of murine models of EM (20). EM also commonly co-occurs with autoimmune disease (73), however, EM has not yet been classified as an autoimmune disease.

While the role of Th1 cells in EM is not entirely understood, Th1 cells are postulated to contribute to disease pathophysiology by promoting chronic inflammation and lesion growth. In a bioinformatic study examining differentially expressed genes in EM patient ectopic tissues as compared to healthy endometrium, Th1 cells, along with memory B-cells, were the top cell types associated with the highest number of hub genes (74). As hub genes are those that are highly connected to other genes and play influential roles in these immune networks, Th1 cells may then play an essential and regulatory role in EM-associated immune dysfunction. Recent literature suggests that Th1 cells may play a particularly influential role in the early stages of EM (75), contributing to the initial inflammatory response and lesion establishment. Indeed, a gene set enrichment analysis (GSEA) by Poli-Neto et al. identified increased Th1 cell signatures in the eutopic endometrium of early stage (I–II) EM patients, suggesting a more active Th1-associated immune response in early disease (51). Though, it should be noted that another study by Wu et al. reported decreased Th1 gene signatures in patient eutopic endometrium compared to controls (50). Others also report that TNFα, a Th1 cytokine, is increased EM patient serum and PF in early disease stages and these levels significantly decrease as EM disease severity increases (21). The inflammatory lesion microenvironment is then thought to switch to an anti-inflammatory, Th2 dominant environment in later stages of EM (51, 75, 76), allowing for the survival and growth of lesions. However, mechanistic studies are required to delineate the precise roles of these immune cell subsets in the chronological development of EM lesions.

Despite aforementioned reports of increased Th1 cells in EM, some report decreased Th1 cells in ectopic tissue (41) as well as no differences in Th1 cell numbers in patient PB as compared to controls (28). Additionally, recent research indicates that Th1 cytokines, IFNγ and IL-2, may serve as independent protective factors for EM (77). Overall, literature surrounding the role of Th1 cells in EM is not entirely standardized and, at times, is conflicting. While definitive markers identifying/characterizing Th1 cells are largely agreed upon, such as IFNγ, T-bet, and CXCR3, there is a lack of standardization in the usage of these markers to conclusively identify Th1 cells.

#### 2.1.2 T helper 2 cells

Th2 cells are a subset of CD4+ T cells that protect against parasitic infection and promote tissue repair through their contribution to the type 2 immune response. Naïve CD4+ T cells differentiate into Th2 cells upon antigenic stimulation in the presence of IL-4 and secrete type 2 cytokines including IL-4, IL-5, and IL-13. IL-4 signaling upregulates the canonical type 2 transcription factor GATA3, which in partnership with STAT5 leads to IL-4 and IL-13 expression (78). Additional transcription factors are required to regulate Th2 programming and are reviewed extensively elsewhere (78, 79). Type 2 cytokines drive both non-hematopoietic (goblet cell hyperplasia, smooth muscle contraction, and fibroblast extracellular matrix production) and hematopoietic cell changes (activation of mast cells, eosinophilia, B cell proliferation and production of immunoglobulin E (IgE), and M2 macrophage polarization). Due to these influences, Th2 cells are associated with disease states that have exacerbated type 2 responses such as asthma/allergic disease and other chronic inflammatory autoimmune diseases including systemic lupus erythematosus (SLE) and IBD. It is however important to note that group two innate lymphoid cells (ILC2s) exhibit a similar secretory cytokine profile as Th2s (IL-5 and IL-13). Thus, delineation is required between these cell types as to their individual and/or combined contribution to disease pathophysiology.

Support for EM as a type 2 inflammatory disease arises from increased detection of type 2 cytokines (IL-4 and IL-10) in patient ectopic tissue (52–54), PF (24, 25), and PB/serum (43, 77), compared to healthy controls. Consistent with this, several studies report increased presence of Th2 cells in EM-associated compartments. Notably, phenotypic characterization of CD4+ cells isolated from PF (22) and PBMCs (80) demonstrated significantly elevated Th2 cells in EM compared to controls, with no apparent influence of disease stage on Th2 cell frequency. Further, intracellular cytokine production of type 1 cytokines, IL-2 and IFNγ, was significantly reduced in CD4+ T cells from EM patient PF compared to controls (23), suggesting a shift to a type 2 response. Contrary to this, Guo et al., found reduced T cell numbers in the PF of stage I and II patients compared to controls, yet no changes in Th2 cell (CD4+CCR4+) frequency (19). However, CD69+ T cells were significantly increased in patient PF compared to controls, with a significant increase in the frequency of CD69+ Th2 cells. Notably, CD69+ T cells were greatest in the PF compared to PBMCs. As CD69 is known as an activation marker, this may suggest an activated T cell phenotype localized to the lesion microenvironment. When examining eutopic tissues in a GSEA study, others identified a decrease in Th2-associated gene signatures in EM patients relative to healthy controls, suggesting a decreased local abundance of Th2 cells (50, 51). This compartment-specific variance highlights a possible disconnect between functional immune phenotypes and transcriptional profiles within the endometrium. Such divergence also highlights important methodological considerations, as markers used to classify Th2 cells differ across publications, highlighting the dynamic nature of these cells yet also potentially leading to discrepancies in reporting. Additionally, many studies report only on total CD4+ T cells without further characterization as to the particular T helper phenotypes.

The EM lesion microenvironment is highly complex with intertwined hormonal, epithelial, stromal, and immune cell cross talk. Local environmental factors at mucosal sites, including epithelial cell-derived alarmin cytokines, such as IL-25, IL-33, and thymic stromal lymphopoietin (TSLP), regulate and fine tune the Th2 response. Indirectly, these cytokines drive Th2 cell differentiation through upregulation of the dendritic cell (DC) co-stimulatory molecule, OX40L (78), and through activation of ILC2s that act on monocytes and macrophages, ultimately regulating Th2 cytokine production and associated downstream functions. These inflammatory cytokines play a large role in Th2-dominant pathological conditions, such as asthma and allergic disease, and have also been found to be elevated in EM. Indeed, our lab has shown that TSLP protein expression is significantly higher in ectopic lesions compared to control endometrium as detected on a tissue microarray of ovarian EM lesions (55). When explored in a mouse model of EM, TSLP treatment increased the PF CD3+ IRF4/T-bet ratio, suggesting an enrichment of Th2 cells with treatment (55). Further, we and others have shown that IL-33 is elevated in EM patient plasma/serum, PF, and ectopic lesions compared to corresponding healthy control samples, which correlates with disease severity (26, 56). This is poignant as pathogenic memory Th2 cells that highly express the IL-33 receptor, suppressor of tumorigenicity 2 (ST2), have been identified in asthma. These cells are known for their high secretion of type 2 cytokines (predominantly IL-5) and amphiregulin which contribute to fibrosis (81). Fibrosis is an important pathological feature of EM thought to contribute to chronic pelvic pain, yet its mechanisms of development remain incompletely understood (82). Thus, mechanistic studies are needed to determine whether Th2 cells are playing a protective or pathogenic role within EM pathophysiology, as there is potential to leverage immunologic therapeutics utilized in other type 2 dominant pathologies.

Finally, the co-occurrence of EM with autoimmune and allergic diseases underscores potential overlapping immunological dysfunction that contributes to the comorbid nature of these conditions (83, 84). A Th2 cell dominant response supports B cell activation and antibody production yet this relationship in EM remains poorly understood. In EM, a positive association between PB and ectopic tissue IL-4 protein and mRNA expression and B cell presence (CD20+CD5+ and HLA-DR+CD20+) has been identified (52), yet Th2 cells were not characterized. Further studies are warranted to explore the relationship between Th2 and B cell activation within EM as Th2 cells may provide a critical link between EM, autoimmune, and allergic disease.

#### 2.1.3 T helper 17 cells

Th17 cells are a subset of CD4+ T cells regulated by the RORγt transcription factor. Known for their production of pro-inflammatory IL-17 and their highly plastic nature, Th17 cells are key regulators of inflammation and immune homeostasis (85), allowing them to contribute to the pathogenesis of various inflammatory and autoimmune diseases (86, 87). The local cytokine milieu shapes Th17 cell phenotype. Namely, IL-23 drives and maintains a “pathogenic” Th17 cell phenotype, producing pro-inflammatory cytokines, such as IL-17, IL-22, and IL-21 (44, 87–89). Pathogenic Th17 cells also co-produce IFNγ, which can in-turn promote Th1 differentiation and inflammation (90). In contrast, “non-pathogenic” Th17 cells differentiate in the absence of IL-23 and produce more anti-inflammatory cytokines, such as IL-10, to regulate/suppress immune responses (44, 91). Indeed, non-pathogenic Th17 cells can play a beneficial/protective role, supporting mucosal barrier integrity and aiding in host defense against bacterial/fungal infections (91).

Dependent on their environment, Th17 cells can also acquire characteristics of other Th subsets, such as Th1 (in presence of IL-12), Th2 (in presence of IL-4), and Tfh (in Peyer’s patches) to induce the development of IgA-producing germinal center B cells (92). Notably, in the presence of TGF-β1, Th17 cells can transdifferentiate into Tregs during an immune response (93). Tregs can also differentiate back into Th17 cells dependent on their environment (94). As a balance of Tregs and Th17 cells ensures immune homeostasis, skewing of this balance can result in the development and/or exacerbation of various chronic inflammatory/autoimmune diseases (94–96). This is particularly relevant to diseases affecting mucosal barrier surfaces (94), such as IBD/IBS, asthma, and EM, as both Th17 and Tregs are commonly found at mucosal surfaces. Ultimately, while it is clear that Th17 cells can act as key modulators of both inflammation and immune tolerance, it is still not completely understood whether Th17 cells completely adopt these various Th lineages, as their transformation is not irreversible, meaning they may still retain characteristics of a Th17 phenotype (97).

The Th17/IL-17 axis has become increasingly researched in the context of EM. Various reports depict increased and/or dysregulated levels of IL-17, Th17 cells, and other key mediators in this axis, within EM patient PF, plasma, menstrual effluent, and/or ectopic/eutopic tissues (27–32, 41, 44–46, 65, 98), as well as within animal models (33, 44, 47, 99), which is reported to be correlated with disease severity (29). Our lab has shown that EM lesions produce IL-17 and patient plasma has significantly elevated IL-17 compared to controls (45). Surgical removal of lesions led to a significant decline in IL-17 levels in plasma, suggesting that EM lesions and associated inflammation may contribute to elevated IL-17 in EM patients. We further depict that IL-17 influences macrophage recruitment and polarization, inducing a M2 phenotype, which aids in lesion development (99). Our lab also depicts dysregulated gene expression of key mediators in the IL-23/Th17 axis in EM ectopic and eutopic tissues, as well as significantly increased IL-23 in patient plasma, compared to controls (44). Recombinant IL-23 treatment of cell lines representative of the endometriotic lesion microenvironment also significantly increased cytokines and growth factors known to play a role in lesion establishment and maintenance (44). Various other reports highlight a dysregulated Treg/Th17 ratio in EM (16), supporting the theory that Th17 cells may skew immune homeostasis to promote inflammation in EM and exacerbate hallmark symptoms of this disease. Ultimately, while Th17 cells and downstream IL-17 have been moderately investigated in EM pathophysiology, a gap in literature remains as to whether/how upstream IL-23 may drive pathogenic Th17 cells in EM to exacerbate disease.

A 2022 study by Jiang et al. reveals Th17 cells to have a significantly different gene expression profile in EM patient PF compared to controls (31). Interestingly, within EM patients, Th17 cells had unique gene expression signatures dependent on disease stage (stages I–II as compared to stages III–IV). The top 10 significantly up-regulated genes were found to encode metabolic proteins, signaling proteins, and cell surface receptors (31). Authors suggest that these results indicate that PF Th17 cells are activated in EM and undergo metabolic reprogramming to provide themselves with sufficient energy. Moreover, the receptor for activated c kinase 1 (RACK1) scaffolding protein was particularly enhanced in EM Th17 cells relative to controls. RACK1 plays a critical role in the regulation of T cell activation, proliferation, and migration (100–102). Indeed, Qiu et al. report that loss of RACK1 increases T cell apoptosis (103). Interestingly, *RACK1* expression was significantly increased in Th17 cells in later stages (III–IV) of EM compared to earlier stages (I–II) (31). Thus, as Th17 cells are associated with EM severity (29), this stage-dependent upregulation of *RACK1* is hypothesized to maintain and/or promote Th17 cell activation and in-turn promote Th17 cell-mediated inflammation in EM, exacerbating disease in a stage-dependent manner (31). A study by Kang et al. also suggests that IL-17 is able to confer a resistance to natural killer (NK) cell-mediated cytotoxicity via activation of ERK1/2 signaling, promoting endometrial cell survival in EM (27).

Recent research on the relationship and association of gut microbiota dysbiosis and EM has also been growing, with numerous reports of gut dysbiosis in EM (104). It is known that shifts in the composition of gastrointestinal and/or urogenital microbiota can regulate differentiation of Th17 and Treg cells to promote either a pathological/pro-inflammatory (Th17) or protective/tolerant (Treg) response (47). Using a baboon model of EM, Le et al. reveal that surgical induction of EM alters mucosal microbiota in both gastrointestinal and urogenital tracts (47). In fact, each stage of EM disease progression was associated with a unique signature of T cell subset dominance and microbial diversity. Namely, authors found immune tolerant Tregs to be associated with microbial diversity during early and late stages of EM progression, while inflammatory Th17 cells were associated with microbial diversity in mid-stages of disease. Though, it should be noted that no sham control was used to confirm results were not due to surgical interventions rather than EM itself. A 2024 study by Li et al. also highlights the correlation between gut microbiota dysbiosis and IL-17 levels in EM, shedding light on how alterations in gut microbiota in EM patients has profound changes in both the intestinal and peritoneal environments (33). Briefly, authors suggest that EM-associated gut dysbiosis compromises intestinal barrier integrity, promotes EM progression, and alters intestinal Th17/Treg balance, subsequently increasing IL-17A levels in patient PF. Due to the influence of gut dysbiosis on Treg/Th17 cell commitment, this can in-turn influence patient susceptibility to IBD (94), which is associated with EM (84). Though, it is unclear whether alterations in immune and microbial phenotypes are a consequence of EM, or if these factors may play a causative role. Collectively, IL-17-producing Th17 cells may promote endometrial cell survival and are reprogrammed under EM-conditions to further promote inflammation and exacerbate disease.

#### 2.1.4 T follicular helper cells

T follicular helper (Tfh) cells are a subset of CD4+ T cells essential for germinal center (GC) B cell maturation and antibody responses (105). Outside of the GC, Tfh cells can fuel cytotoxic CD8+ T cell responses (106) and contribute to tertiary lymphoid structures (TLSs) in chronic inflammation (106), such as in EM. Tfh cell differentiation requires T cell receptor (TCR) and inducible T-cell co-stimulator (ICOS) activation, often provided by DCs, in the presence of IL-6, which upregulates Bcl6 and CXCR5 expression, enabling Tfh cell trafficking to inflammatory structures, including secondary lymphoid organs (SLOs) and ectopic TLSs, through the receptor ligand CXCL13 (105). Once in TLSs, B cells replace DCs as the primary antigen presenting cell and Tfh cells maintain B cells via IL-21 and further produce CXCL13 to recruit additional T and B cells (106). Tfh cells are often characterized as CD4+CXCR5+Bcl6hi (107). Though, Tfh cells exhibit plasticity, with subsets resembling Th1, Th2, and Th17 cells (Tfh1, Tfh2, Tfh17) (105), which is of interest due to aforementioned roles of these Th cells in EM pathophysiology. Tfh cells also play a key role in various autoimmune diseases including SLE and Sjögren’s syndrome, in which there is extensive autoantibody production (105). It is suggested that their ability to support B cell antibody production may contribute to autoantibody development (108). As EM is associated with autoantibodies (109), Tfh cells may play a similar role in EM pathophysiology.

Tfh cells also play a crucial role in the formation and function of TLSs, which are organized immune cell aggregates known to form in cases of chronic inflammation and/or persistent antigenic exposure (110). This is of interest as our group recently discovered the presence of TLSs across varying phenotypes of EM (110). While T cell markers (CD3 and CD8) were used, Tfh cells were not specifically highlighted by the multiplex immunofluorescence panel used to classify TLSs within our study (110). Thus, continued characterization of EM-associated TLSs is critical to determine the contribution of diverse immune phenotypes to these dynamic structures.

While the role of Tfh cells is understudied in EM, literature depicts significant differences in local proportions of Tfh cells in EM tissues compared to controls (111). Indeed, Wu et al. revealed an enrichment of Tfh cell-associated gene signatures in eutopic tissues of patients compared to healthy controls, suggesting an increased abundance of local Tfh cells (50). Notably, a 2022 study found Tfh cells were significantly reduced in EM lesions compared to matched eutopic tissues of patients (57). Moreover, Tfh cells were correlated with aquaporin 1 (AQP1) and ZW10 binding protein (ZQINT), two proposed diagnostic markers of EM, and showed positive correlations with Tregs and activated NK cells, but negative correlations with M2 macrophages. Others report significantly increased Tfh cells in EM compared to healthy controls (80, 112). Though, these authors observed Tfh cell proportions in PB (80), underscoring the need to better define Tfh cell presence within lesion tissue.

#### 2.1.5 T regulatory cells

Tregs contribute to immune homeostasis by suppressing exaggerated immune responses and promoting immunological self-tolerance. Consequently, reduced Treg cell function is implicated in autoimmune disease (113). Tregs carry out their effector functions via direct cell-cell interaction, cytotoxic action, and release of suppressive cytokines, including IL-10, TGF-β1, and IL-35 (114). These cytokines can act to inhibit effector T cell activation, while granzyme and perforin produced by Tregs can act to kill effector T cells (115). IL-10 can also function to inhibit DC maturation, further contributing to immune tolerance (116). Moreover, the immunosuppressive actions of Tregs are primarily regulated by FoxP3, a transcription factor crucial for Treg cell differentiation and function (117). Efficient Treg function requires the expression of *FOXP3*, in addition to other signature Treg genes, such as *IL2RA* (CD25) and *CTLA4 (*118). Three distinct subpopulations of Tregs exist in humans: resting suppressive Tregs (CD45RA+FoxP3^low^), high-activated suppressive Tregs (CD45RA-FoxP3^high^), and cytokine-secreting, non-suppressive Tregs (CD45RA-FoxP3^low^) (118). In mice and humans, Tregs may also be categorized by origin/developmental context into natural Tregs, developed in the thymus, and induced Tregs, differentiated from naïve CD4+ T cells in the periphery/outside the thymus (119).

Tregs may contribute to EM pathophysiology in part through the prevention of adequate immune surveillance and lesion clearance by other immune cell types, allowing for lesion establishment and growth. Alterations in Treg homeostasis, both systemically and locally within ectopic and eutopic endometrium, have been noted in EM (116). However, differing methodology and characterization of Treg cells has resulted in variable results. A single-cell ligand-receptor analysis revealed upregulation of *CTLA4*, which was interacting with *CD86*, in ectopic lesions of stage II–IV EM patients, indicative of highly activated immunosuppressive Tregs (58). Thus, Tregs may facilitate immune evasion in ectopic tissues, promoting local lesion persistence. This is further supported by findings of Treg density in patient PF being correlated with EM stage and severity (120). In a flow cytometric analysis of PB and PF from EM patients, an increase in Tregs (CD25^high^FoxP3+) was noted in PF compared to controls, while a decrease in Tregs was noted in PB (34), suggesting altered Treg compartmentalization in EM. Khan et al. similarly reported increased Tregs in patient PF, though, no significant differences were found in PB (30). Through IL-10-mediated inhibition of DC maturation (58), Tregs may also contribute to the tolerogenic, immature DC phenotype observed in peritoneal lesions. Thus, Tregs may support immune evasion observed in EM, facilitating lesion establishment and preventing effective clearance.

Treg secretion of soluble fibrinogen-like protein 2 (sFGL2) appears to promote an anti-inflammatory M2 phenotype, which supports tissue repair/remodeling (121). Notably, sFGL2 levels are increased in EM patient PF, which may locally promote this alternatively activated M2 phenotype within the lesion microenvironment (121). This suggests a positive feedback loop between Tregs and macrophages that perpetuates EM progression. As M2 macrophages play a crucial role in EM-associated fibrogenesis, Tregs may also indirectly contribute to fibrosis (122), a key feature of EM linked to pain and infertility (82). However, contradictory findings exist. Tanaka et al. reported a significant reduction in activated Tregs (CD45RA-FoxP3^high^) in both ectopic and eutopic EM tissues compared to controls (35), which was not observed in the PF or PB. Though, as only ovarian endometrioma tissues were utilized, this finding may be specific to this type of EM. This study also used a mouse model of EM, whereby Tregs were selectively depleted in FoxP3^DTR^ (diphtheria toxin receptor) C57BL/6 mice (35), resulting in increased systemic (PB) and local (PF, lesions) levels of inflammatory cytokines and angiogenic factors, as well as increased lesion number and weight compared to controls. As the lack of Tregs facilitated EM progression, this further supports aforementioned theories of an initial pro-inflammatory, Th1/Th17-dominant microenvironment which transitions into a later anti-inflammatory, Th2/Treg-dominant microenvironment to allow for EM lesion growth/maintenance.

Tregs also play a crucial role in regulating angiogenesis, another key hallmark feature of EM. Indeed, their angiogenic role is well-known during embryo implantation and pregnancy, though, whether they play an anti- or pro-angiogenic role is highly tissue and context specific. In an ovarian cancer study, Tregs were recruited to tumor sites via CCL28, where they contributed excess VEGFA and promoted tumor angiogenesis (123). Notably, Treg depletion in mice using an anti-CD25 antibody resulted in a significant reduction in tumor microvascular density within intraperitoneal tumors (123). Moreover, in another study of abortion-prone mice, adoptive transfer of Tregs improved spiral artery remodeling and placental development, influencing vascularization (124). Thus, Tregs may function in a similar fashion in EM, promoting angiogenesis and lesion establishment. This is supported by *in vitro* evidence in which co-culture of primary human endometrial stromal cells (ESCs) and monocytes increased CCL17 and CCL22 production, promoting Treg recruitment, enhanced immunosuppressive function, and increased TGF-β1 secretion, which may collectively support angiogenesis in endometrial cells (125). However, this study had limitations, such as using eutopic ESCs rather than ectopic cells. Ultimately, further research is required to elucidate the exact role of Tregs in EM-associated angiogenesis.

Our lab and others have demonstrated increased IL-33 in EM, which may drive disease progression by perpetuating inflammation, angiogenesis, and lesion proliferation (26, 56, 126). IL-33 also influences Treg cell fate and function, which may then impact EM. Indeed, in a mouse study on skin transplantation, IL-33-stimulated Tregs displayed potent anti-inflammatory activity (127). Further, in the presence of TGF-β1, Tregs can be differentiated from naïve CD4+ T cells through the IL-33/ST2 pathway, via regulation of FoxP3 expression (128). IL-33 binding to ST2 both promotes Treg fate and increases ST2 expression, creating a positive feedback loop for Treg expansion (127). Others have also shown that IL-33 promotes functional stability of Tregs *in vivo*, allowing for enhanced immune evasion (129). Ultimately, one may draw parallels from these findings, suggestive that a similar mechanism may exist in EM whereby IL-33 may mediate Treg stability, allowing for evasion of EM lesion clearance by the immune system.

### 2.2 CD8+ T cells in endometriosis

CD8+ T cells play a key role in immune surveillance and produce pro-inflammatory cytokines, including TNFα and IFNγ, to facilitate immune cell recruitment and sustain chronic inflammation, respectively (130, 131). Cytotoxic CD8+ T cells are a critical component of the adaptive immune response, targeting and eliminating infected, malignant, and foreign cells. In healthy states, cytotoxic T cells control viral infections and provide tumor surveilance (132). However, their activity is context dependent. While vital for clearing infections, they can also promote inflammation and tissue/joint damage, as observed in autoimmune diseases, such as RA (133, 134). Notably, CD8+ T cells exhibit functional plasticity (62, 135), with research identifying an IL-17-producing CD8+ subset known as Tc17 cells, which are increased in EM patients, potentially driven by high IL-23 levels (44, 80, 136). This plasticity allows them to modulate immune responses, contributing to both protective immunity and immunopathology.

The involvement of CD8+ T cells in the initial establishment of EM lesions remains under investigation. Literature depicts an increased proportion of CD8+ T cells in EM lesions when compared to matched eutopic endometrium from EM patients and/or healthy eutopic endometrium (59). Overexpression of CD8+ T cells in peritoneal EM lesions, as compared to both eutopic endometrium and healthy control peritoneum, has been established (60, 61, 137). However, in a study aiming to investigate cellular heterogeneity in EM, the proportion of CD8+ T cells was reduced in EM lesions, with the cytotoxicity of ectopic T cells being depressed (62). Interestingly, CD8 inhibition in an EM mouse model resulted in visually larger lesions, while mice supplemented with CD8+ T cells showed reduced weight in EM lesions (62). Evidence suggests that these cells play a significant role in the maintenance and progression of established lesions (62). Indeed, a 2023 systematic review highlighted alterations in the function and number of CD8+ T cells within EM, finding consistent indications of CD8+ T cell enrichment within EM lesions, indicating their likely contribution to lesion persistence (137). Though, no consistent, significant alterations were identified in CD8+ T cell populations within PB, PF, or eutopic endometrium samples from EM patients/controls (137).

In the context of EM, disease progression is marked by impaired immune surveillance and ineffective clearance of ectopic endometrial cells within the peritoneal cavity, ultimately promoting lesion establishment. Chronic antigenic stimulation, as observed in persistent infections and tumors, drives the development of CD8+ T cell exhaustion (135, 138). Although persistent exposure to non-self-antigens is the most studied cause of T cell exhaustion, exhaustion can also arise in response to self-antigen presentation, particularly in the context of chronic inflammation (139). Under steady-state conditions, classical tolerance mechanisms such as deletion and anergy maintain self-reactive T cells in check (140). However, when self-antigens are presented in an inflammatory environment, exhaustion may be observed as an additional regulatory mechanism (139, 141). In EM, persistent self-antigen exposure, coupled with inflammation and immune activation, may promote CD8+ T cell exhaustion as a means of limiting tissue damage. While this response can help suppress excessive inflammation, it may also impair the cytotoxic capacity of CD8+ T cells, reducing their ability to clear ectopic endometrial debris and developing lesions (135, 138).

Exhausted CD8+ T cells exhibit impaired cytotoxicity due to dysregulated perforin and granzyme expression and sustained expression of inhibitory receptors, notably PD-1, which enforce their dysfunctional state (135, 138). These cells undergo a progressive loss of effector functions, beginning with compromised/lost production of IL-2, followed by TNF−α and IFN−γ, ultimately resulting in terminally exhausted cells with limited proliferative capacity and reduced persistence (142). A study by Schmitz et al. demonstrated that CD8+ T cells isolated from menstrual effluent of EM patients showed significantly lower perforin expression compared to controls, indicating a reduction in cytotoxic potential (66). The reduced number of perforin-positive CD8+ T cells may be explained by incomplete CD8+ T cell differentiation, potentially caused by inhibitory cytokine signaling (e.g. IL-10 and TGF-β) or cytotoxic activation leading to granule exocytosis (66). Given that the most widely accepted theory for the etiology of EM involves retrograde menstruation (143), it is possible that the lack of perforin-positive CD8+ T cells in the menstrual effluent allows for lesion establishment. Similarly, Iwasaki et al. found a reduction in cytotoxic T cells in both the PB and PF of EM patients, further supporting the notion of compromised immune function in this condition (36).

In addition to their cytotoxic functions, CD8+ T cells secrete key cytokines, including IFNγ, TNFα, and lymphotoxin-α (LT-α), which play crucial roles in modulating host defense responses (135). IFNγ has been observed to be significantly upregulated in the PF of EM patients relative to fluid from healthy controls (24). A study conducted by Wu et al. found that in response to IFNγ stimulation, intercellular adhesion molecule-1 (ICAM-1) expression was elevated in cultured cells isolated from both eutopic endometrium and peritoneal EM lesions (144). ICAM-1 is a prominent cell adhesion molecule displaying widespread distribution in the human endometrium, regulating leukocyte trafficking into the tissue and linking adhesive interactions with epithelial and endothelial function (145). Various studies have shown a significant increase in ICAM-1 in the serum, PF, and lesions of EM patients, relative to controls (144, 146, 147). Together, these may indicate that an increase in IFNγ may promote the adhesive and invasive activity of ectopic lesions, leading to EM progression (144, 146). Elevated TNFα levels are commonly observed in both acute and chronic inflammatory conditions, including EM and numerous autoimmune diseases. Notably, Liu et al. found that increased localized expression of TNFα correlates with the presence of active (vascular, inflammatory, proliferative (148)) EM lesions (149). Similarly, LT-α, a member of the TNF superfamily, is implicated in the pathogenesis of various chronic inflammatory diseases. In advanced stages of EM, serum LT-α levels are significantly elevated compared to healthy controls, underscoring its potential role in disease progression (150).

Cross-sectional and case-controlled studies report a significant association between EM and increased incidence of RA (84, 151–153). Patients with active RA exhibit increased unstimulated CD8+ T cells expressing TNFα and granzyme B (154), as well as increased serum TNFα relative to healthy controls (155). As TNFα is also elevated in EM serum, this suggests a potential shared inflammatory mechanism, where CD8+ T cell-derived TNFα may recruit pro-inflammatory cells that amplify cytokine-drive inflammation, exacerbating EM. CD8+ T cell dysregulation may also contribute to EM-associated infertility (137), as impaired cytotoxic function can foster a pro-inflammatory environment, adversely affecting reproductive processes (137). While the exact role of CD8+ T cells remains unclear, there are correlations between this cell type’s associated cytokines and infertility (50). This aspect is extensively covered elsewhere (137).

## 3 Other emerging and underexplored T cell subsets in endometriosis

### 3.1 T helper 9 cells

Th9 cells have emerged to characterize CD4+ T cells that secrete IL-9. Previously IL-9 production was included under the banner of Th2 cell secretions, however, studies have shown that CD4+ IL-9 producing cells are independent in both their secretory and transcriptional profiles from the other T-helper subsets (156, 157). Th9 cells can be differentiated through two pathways, either from naïve CD4+ T cells in the presence of IL-4 and TGF-β1, or from Th2 cells in the presence of TGF-β1. For this reason, Th9 cells are thought to be a subset of Th2 cells (158). Due to the requirement for IL-4 and TGF-β1 in differentiation, these cytokines complimentary surface receptors, IL-4R and TGF-β RII, are commonly used for phenotypic characterization of Th9 cells (157). As with Th2 cells, IRF4 is an important transcription factor in Th9 development, in addition to PU.1, BATF3 (156), and PPARγ (158). Th9 cells function to enhance protective immunity at mucosal surfaces through IL-9 on mast cells, epithelial cells, and smooth muscle cells. Adversely however, Th9 cells have been identified in asthma, inflammatory gut reactions, and autoinflammation, with a particular role in fibrosis due to IL-9 production (159).

In EM, IL-9 has been identified as significantly elevated in the PF (37) and plasma (160) of patients compared to controls. However, the primary source of IL-9 remains to be interrogated. Tarumi et al., identified significantly higher presence of Th9 cells (CD4+IL-9+) in the PF of patients with ovarian EM, but did not find a corresponding increase of IL-9 in the PF compared to healthy controls (38). A critical relationship that requires continued interrogation in EM is that of mast cells and Th9 cells. Our lab has identified mast cells as contributors to EM pathophysiology (161). In a mouse model of EM, adoptively transferred Th9-like cells induced no alterations to mast cell numbers (162). However, Th9 cells infiltrated the lesion and promoted reduced proliferation and increased lesion-associated inflammation through upregulation of IL-1α. In pathological contexts such as asthma, IL-9 drives proliferation and recruitment of mast cells to sites of lung inflammation (38). Thus, whether mast cell and Th9 cell cross talk shapes the EM lesion microenvironment and associated alterations remain to be explored.

### 3.2 Memory T cells

As aforementioned, EM is characterized by high recurrence rates post excision surgery (8, 11). Persistent immunological dysfunction and chronic inflammation may contribute to recurrence despite surgery or medical treatment (163). Notably, memory T cells, characterized by their long lifespan and enhanced recall responses, may play a key role in driving and maintaining immune dysfunction in EM recurrence. These memory T cells can be either CD4+ or CD8+ and are mainly divided into three subpopulations: central memory T cells (TCM), effector memory T cells (TEM) and tissue resident memory T cells (TRM), depending on their ability to migrate within SLOs and non-lymphoid tissues (NLT) (164, 165) through the expression of CCR7 and CD62L (166, 167). Like TEM, TRM cells do not express SLO homing markers, but do exhibit prolonged expression of the activation marker CD69, which blocks S1P1-mediated tissue egress (168), and retention surface proteins to their tissue of residency including CD49a and CD103 (169, 170). TCM and TEM cells exhibit functionally distinct responses to TCR stimulation. TEM primarily release effector molecules including IFNγ and perforin, enabling rapid immune response. In contrast, TCM predominantly secrete IL-2, supporting T-cell proliferation and survival and can differentiate into effector T cells and TEM (171, 172). At local inflammatory sites, TRM cells play the role of front-line defense, as they effectively detect pathogens and exert typical memory functions including cytotoxic granzyme B and IFNγ secretion (173, 174).

Memory T cells, including TRM, have been extensively studied in various mucosal and non-mucosal tissues in both healthy and disease contexts (175–177). However, these memory cell types are surprisingly overlooked in the upper female reproductive tract and in the context of EM. Some literature suggests differing levels of CD8+ memory T cell subsets within the eutopic endometrium and PB of EM patients compared to controls, though, data overall appears to be inconsistent (137). For instance, two GSEA studies depicted CD8+ TEM gene enrichment scores were increased in the eutopic endometrium of EM patients compared to controls (51, 63). While, in contrast, another GSEA evaluation demonstrated decreased TEM and TCM scores (50). Though, this study did not appear to delineate TEM or TCM subsets by CD4/CD8 expression. Another study found elevated levels of peripheral CD8+ TCM but reduced number of terminally differentiated CD8+ TEM cells in EM patients (178). Ultimately, further studies need to address these controversial results and elucidate the pathophysiological roles of memory T cells in EM.

### 3.3 Gamma/delta T cells

Conventional alpha beta (αβ) T cells are the focus of most reports on the role of T cells in EM, while gamma delta (γδ) T cells are often overlooked. γδ T cells possess a different TCR pattern consisting of γ and δ chains and generally lack both CD4 and CD8 expression on their cell surface, distinguishing them from their αβ counterparts (179). γδ T cells can recognize a wide range of antigens without relying on major histocompatibility complex (MHC)-dependent antigen presentation (180, 181), serving as a linking arm between the innate and adaptive immune response. In humans, Vδ1+, Vδ2+, and Vδ3+ are the three main subsets of γδ T cells with Vδ2+ T cells most prevalent in PB and Vδ1+ and Vδ3+ T lymphocytes predominantly located in tissues (182). Unlike human γδ T cells categorization, their murine counterpart is divided into six distinct subsets (Vγ1, Vγ2, Vγ4, Vγ5, Vγ6, Vγ7) based on their γ-chain locus (183). Despite the difference in their subpopulation determination, human and murine γδ T cells share common features such as early fetal development, restricted TCR diversity, and antigen recognition by butyrophilins (184, 185). γδ T cells are widely dispersed throughout mucosal tissues, including the female reproductive system, where their numbers may vary in response to hormonal shifts (182). About 5–10% of human uterine T cells and 60–80% of mouse uterine T cells have γδ T cells spread throughout the endometrium (186–188). Estrogen receptors (ERs) are highly expressed by γδ T cells in the uterus, which promote cell expansion and trigger the release of CXCR3 and IL-17A in response to estrogen signalling (186, 189, 190). Thus, the dysregulated immunological milieu that characterizes estrogen-driven diseases, such as EM, may be significantly influenced by γδ T cells.

Hudecek et al. confirmed the presence of γδ T cells in both PF and PB of EM patients (48). Specifically, authors observed a significant decrease in circulating Vδ1+ T cells (PB) in EM as compared to healthy controls, whereas circulating Vδ2+ T cell numbers remained unchanged. Another study found that Vδ1+ T cells in EM patient PB exhibited a heightened activation state and possible signs of exhaustion due to elevated PD-1 levels (80). In contrast, Vδ2+ T cells in EM patient PB had reduced activation and cytotoxic capabilities. Peripheral Vδ1+ T cells were also positively correlated with EM disease severity and presence of pain symptoms, while peripheral Vδ2+ T cells were negatively correlated with these aspects of EM. Overall, as authors found reduced frequencies of Vδ2+ in EM PB, as well as higher frequencies of PD-1+Vδ1+ and PD-1+Vδ2+ T cells in EM compared to controls, they suggest that γδ T cells may develop an exhausted phenotype after activation due to the prolonged chronic inflammation in EM (80). While further research is necessary to confirm this, these studies provide evidence of an imbalance of Vδ1+ and Vδ2+ T cells in EM, which may be associated with disease severity and pain symptoms.

In the context of endometrial and ovarian cancers, γδ T cells have been shown to infiltrate tumors and contribute to a protective response through their tumor-killing activity. However, this effect can be suppressed by factors, such as EphA2, which inhibit their tumor lysis function (48, 191). The specific functions of γδ T lymphocytes in EM are still largely unknown, despite evidence of their varied participation in the uterine endometrium and several gynecological cancers. As γδ T cells are one of the most abundant T cell populations at mucosal surfaces, including the uterus, where they enact unique MHC independent cytotoxic response and recognition of stress ligands, future studies are required to understand their specific contributions to EM, as these cells form a unique bridge between innate and adaptive immunity. More specifically, given their abundance in the uterus and Sampson’s theory of retrograde menstruation (143), research should aim to assess whether γδ T cells are maintaining similar roles in eutopic and ectopic tissues.

### 3.4 Natural killer T cells

Natural killer T (NKT) cells are a unique T cell subset with a highly restricted, invariant TCR that recognizes both self and foreign lipid-based antigens presented by CD1d. Thus, they are commonly referred to as invariant NKT (iNKT) cells, which can be CD4+/-. Human iNKT cells predominantly express CD8α, while mouse iNKT cells generally lack CD8 expression (192, 193). CD4+ iNKT cells share similar transcriptional and subpopulation features with conventional CD4+ T cells (194). Upon activation, iNKT cells secrete a variety of cytokines and chemokines, including Th1 cytokines (IFNγ, TNF), Th2 cytokines (IL-4, IL-10, IL-13), IL-2, IL-3, TGF-β (195–197), and IL-17 specifically from NK1.1 lacking subsets (198). iNKT cells are critical for successful implantation and maternal-fetal tolerance, by proliferating in the decidua (endometrium) and recognizing CD1d-presented fetal glycolipids (199, 200). Indeed, abnormal NKT levels in human and mouse decidua are linked to recurrent miscarriages (201–203). Though, the role of NKT cells is still largely underexplored in EM pathophysiology.

Reports demonstrate that the overall number of iNKT cells and double-negative (DN) iNKT cells are decreased in EM patient PB, particularly during the secretory phase, compared to healthy controls. Moreover, patients with severe dysmenorrhea observed an increase in CD4+ iNKT cells expressing IL-17 (49). These findings align with a previous study by Guo et al. that reported a decrease in NKT cells number in both PB and PF of EM patients compared to healthy individuals. Interestingly, higher levels of IFNγ and IL-4 levels were positively associated with the NKT cell numbers while an inverse correlation was observed with disease stage (39). Moreover, a transcriptome meta-analysis by Poli-Neto et al. revealed NKT gene signatures are increased in the eutopic endometrium of EM patients, most predominantly stage I–II patients, as compared to healthy controls, suggesting an increased local abundance of NKT cells in early disease (64). However, potential contributions of NKT cells in EM pathophysiology remain largely unexplored.

### 3.5 Double negative T cells

DN T cells are characterized by expression of TCRαβ but absence of CD4/CD8 co-receptors and NK T cell markers. These cells are thought to originate from either thymus exit or the loss of CD8 expression in the periphery after TCR and cytokine stimulation (204–207), residing in PB, SLOs, and a variety of tissues (tissue-resident DN T cells), including liver, lungs, skin, intestine, and genital tract. Circulating DN T cells can also migrate to infiltrate non-lymphoid tissues (NLTs), contributing to tissue-specific immune responses (208). Like Th17 cells, DN T cells exhibit functional plasticity, whereby they can act as a major source of IL-17, contributing to chronic inflammation and tissue damage in autoimmune disease (209), or can produce a marked level of IL-10 and exert regulatory roles as a specific peripheral subset of DN Tregs (210). This plasticity is demonstrated in cancer, where DN T cells can be tumor-promoting or -suppressing depending on cancer type and the tumor microenvironment, with their presence noted in both PB and solid tumors (lung, liver, glioma, pancreatic cancer) (211–214). In an autoimmune-prone *lpr* (fas-deficient) murine model, DN T cells exhibited immunosuppressive activity via IL-10 secretion (215). Therapeutic approaches targeting DN T cells show promise in treating EM-associated comorbidities, including cancer and autoimmune conditions (216, 217), suggesting potential for EM-specific treatment.

### 3.6 Mucosal-associated invariant T cells

Mucosal-associated invariant T (MAIT) cells are unconventional T cells with a highly conserved TCR repertoire (218), primarily found in mucosal tissues but also present in PB. MAIT cells are identified by the TCR α-chain (Vα7.2–Jα33 in humans, and Vα19–Jα33 in mice) and restricted TCR β-chain repertoire (219, 220), with high expression of C-type lectin CD161 in humans. These cells contribute to both innate and adaptive immunity and are implicated in autoimmune and immune-mediated diseases (218). Upon IL-18-dependent activation, MAIT cells produce proinflammatory molecules, including IL-17, IFNγ, TNF, and cytotoxic factor granzyme B (221), with IL-12, IL-15 and type I interferons further enhancing activation (222). In autoimmune conditions, including MS (223), IBD (224), and RA (225, 226), circulating MAIT cells are reduced in patient PB, but markedly increased in affected tissues, reflecting their migration to inflamed sites. Decreased circulating MAIT cells may also be attributed to prolonged activation leading to cell death, as MAIT cells expressed increased exhaustion markers (CD38, PD-1) and apoptosis indicators (FAS, intracellular active caspase 3) (218).

MAIT cells migrate and accumulate in inflamed tissues via chemokine receptors (CCR5, CCR6 and CXCR6), integrins (α4β1 integrin, CD49a and CD103), and adhesion molecules (PSGL1 and sLex). Notably, IL-18, the major activator of MAIT cells, is enriched in both the PB and PF of EM patients (227). A pilot study measured several factors crucial to MAIT cell functioning and observed enriched levels of IL-8, IL-12 and in the PF of EM patients compared to healthy controls. Interestingly, MAIT cells were increased in both PF and PB of EM patients. Specifically, CD4+ and CD8+ MAIT populations expanded, while DN MAIT cells were reduced. CD8+ MAIT cells in EM patients were highly activated by expressing more robust levels of CD38, while lower DN MAIT cell number was attributed to elevated PD-1 expression (40). However, no study has yet explored the functional role of MAIT cells in EM.

## 4 Chemokine-driven regulation of T cell trafficking to the endometriosis lesion microenvironment

T cell trafficking to the EM lesion microenvironment is an active area of research that is still not fully understood, though, several key findings have emerged. A study by Kim et al. illustrated that PF from EM patients significantly increased the release of CXCL10 by CD4+ T cells isolated from donor PBMCs, as compared to control PF (228). As CXCL10, also known as IP-10, is well recognized for its role in recruiting activated T cells to sites of inflammation/tissue damage (229, 230), CD4+ T cells within the EM lesion microenvironment may be major drivers of CXCL10 production, and in-turn, play a key role in promoting T cell recruitment to the endometriotic lesion. Another study by Manabe et al. found that both CXCL16 and its receptor, CXCR6, were expressed by endometriotic epithelial and stromal cells, but not normal ovarian stroma (231). As CXCL16 facilitates T cell migration in other contexts, such as psoriasis (232) and RA (233), via its receptor CXCR6, this chemokine may play a similar role in EM. Authors also found that CXCL16 induced IL-8 (CXCL8) production in ESCs *in vitro (*231). IL-8 is well-known to be involved in EM-related inflammation and fibrosis (234), and thus, CXCL16 may further exacerbate the lesion milieu via IL-8. Moreover, others demonstrate that CXCL16/CXCR6 mediates the recruitment of pathogenic Th17 cells and IL-17A+ γδ T cells into atherosclerotic lesions (235). Thus, CXCL16/CXCR6 may also facilitate T cell migration in EM, however, further research is required to examine these chemokines specifically in this disease context.

Th17 cells express CCR6 in EM tissues (236) and PF (31). Thus, CCL20 (CCR6 ligand), which is expressed in endometriotic epithelial and stromal cells, can cause the selective migration of Th17 cells in EM via CCR6 (236). The frequency of CCR6+ Th17 cells are also significantly increased in advanced EM compared to mild EM (31), suggesting increased susceptibility to CCL20-mediated migration to the lesion microenvironment in more advance cases of disease, whereby Th17 cells may further exacerbate key hallmark features of EM. CCL20 is also known to stimulate the migration of Treg cells in EM (237). Indeed, when CCL20 is neutralized, this chemotactic activity is inhibited (237). Moreover, as detailed earlier, a study by Wang et al. depicts that ESCs co-cultured with monocytes produce high levels of CCL17 and CCL22 and that these chemokines cause the recruitment of Treg cells (125). Thus, while various factors may be at play, these chemokines may be considered as therapeutic targets to regulate Th17 (CCL20) and Treg cell (CCL17/20/22) migration to the EM lesion microenvironment.

## 5 Diagnostic and therapeutic implications: an overview of current T cell targeted strategies and upcoming therapeutic targets

Given the central role of T cells in EM disease pathophysiology and emerging evidence successfully targeting T cells in other autoimmune and chronic inflammatory conditions, there is a growing interest in exploring how T cells could be harnessed for therapeutic purposes in EM. A recent study by Zhang et al. identified ectopic endometrial T cells as the primary source of active IL-16 (98), a key initiator of EM-associated inflammation. Indeed, IL-16 knockout mice exhibited reduced inflammation and lesion growth (98). As iron overload is observed in EM (238), authors recapitulated these conditions *in vitro* and found this drives the caspase-3-GSDME-mediated pyroptosis axis in ectopic endometrial T cells, initiating IL-16 activation and release. Targeting this pathway, authors developed Z30702029, a GSDME-NTD inhibitor, depicting that Z30702029 alleviated EM lesion development and associated pain *in vivo (*98). However, this has yet to be examined in human patients. Another study by Park et al. examined the therapeutic potential of S-Aallyl-L-cysteine (SAC) in EM, a large component of aged garlic extracts, using a mouse model of EM and found that SAC treatment significantly reduced lesion growth (239). Moreover, treatment was shown to alter splenic CD4+ and CD8+ T cells and inflammatory cytokine production within samples from spleen and EM lesions (239).

While PD-1 and PD-L1 proteins have been extensively studied in cancer due to their regulation of immune tolerance within the tumor microenvironment (240), the role of the PD-1/PD-L1 pathway in EM remains unclear. Sobstyl et al. recently discovered that expression of PD-1 and PD-L1 is significantly increased on circulating T and B lymphocytes within EM patient PB as compared to controls (241). This was also reflected when analyzing serum concentrations of soluble (s)PD-1 and sPD-L1 forms in EM patients (correlated with stage) as compared to controls. Another study by Abramiuk et al. found significantly increased levels of CTLA-4+ T cells in advanced cases of EM compared to patients with less advanced disease (242). This is of interest as CTLA-4, a membrane receptor that facilitates lymphocyte anergy, may play a role in the dysregulated immune response within the endometriotic microenvironment. Thus, further research is required to comprehensively investigate the role of PD-1/PDL-1 and CTLA-4 in EM and their potential for therapeutic targeting. Another study by Tian et al. aimed to examine the impact of T cell Ig and mucin domain-containing molecule-3 (TIM-3), an immunomodulatory molecule found on the surface of Th1 cells which mediates Th1 cell apoptosis, in EM (243). Using both *in vitro* and *in vivo* studies, authors depict that TIM-3 promotes EM lesion proliferation via the brain-derived neurotrophic factor (BDNF)-mediated PI3K/AKT axis, providing another possible therapeutic target of interest in EM.

Tregs are integral in maintaining immune tolerance and modulating the immune response, and thus, targeting Treg activity offers a potential avenue of interest in EM. Indeed, a study by Li et al. found significantly increased Tregs, along with overactivation of estrogen-ERα, within EM patient PF (244). Estrogen-ERα was also found to promote Treg expansion and cytokine production, namely IL-10 and TGF-β1 cytokines, which promote the survival and growth of EM lesions (120). Authors further showed that treatment with SCM-198 (a synthetic derivative of leonurine) significantly reduced number and expansion of peritoneal Tregs, decreased levels of immunosuppressive cytokines (IL-10 and TGF-β1), and attenuated estrogen secretion by lesions (244). Moreover, using a Treg-depleted mouse model of EM, adoptive transfer of Tregs significantly reduced number, volume, and weight of EM lesions (245), as well as reduced mRNA expression of Th1-, Th2-, and Th17-related cytokines, such as IFNγ, IL-4, and IL-17, and pro-inflammatory IL-6 within EM lesions of Treg-depleted mice.

As aforementioned, almost all patients with EM report at least 1 comorbid disorder (8), of which, a large majority appear to be driven by T cell dysfunction. A study by Shigesi et al. suggests an association between EM and autoimmune diseases, including systemic lupus erythematosus, Sjögren’s syndrome, RA, autoimmune thyroid disorder, celiac disease, MS, and IBD (84). Interestingly, the IL-23/Th17/IL-17 axis plays a role in each of these EM-associated autoimmune diseases (246–250), suggesting a possible overlapping/shared pathogenic pathway. This is of high interest for therapeutics, particularly as IL-23- and IL-17-targetted therapeutics (several monoclonal antibodies) have already undergone clinical trials and are commonly used clinically to treat cases of psoriasis, ankylosing spondylitis, arthritis, and Crohn’s disease (251). Moreover, due to the known role of IL-17 in EM-associated infertility (252), this pathway is of high interest for therapeutic targeting. Ultimately, it is speculated that particular autoimmune diseases may be largely facilitated by a dysregulated IL-23/Th17/IL-17 axis, and as this now also being depicted in EM, blocking IL-23 and/or IL-17 systemically/locally in EM patients may suppress lesion development/progression as well as associated infertility.

EM and asthma are also often comorbid and have a positive association (253). While this association is not completely understood, this may be due to an exaggerated type 2 immune response/mast cell activation (161). Indeed, it is postulated that the exacerbated type 2 response (Th2-dominant) in EM downregulates the pro-inflammatory type 1 response (Th1-dominant) and promotes aberrant wound-healing leading to lesion maintenance and progression. Thus, interruptions in these pathways could be beneficial to lesion clearance. As previously highlighted, epithelial cell-derived cytokines (IL-33, IL-25, TSLP) act as instigators of the type 2 immune response and both these cytokines and downstream cytokine products (IL-4, IL-5, IL-13) are elevated within EM. This provides an opportunity for therapeutic targeting of upstream and/or downstream components to dampen the type 2 response, and in asthma, such biologic therapeutics are already in clinical trials or approved for patient use (254–256). Given the overlapping immunological pathways between these chronic inflammatory conditions, existing therapeutics could be leveraged for EM patients. For instance, retrospective analyses of EM patients with asthma, or other associated comorbidities, could be conducted to determine whether these existing therapeutics also impact EM symptomology or disease progression. Such findings could inform a personalized medicine approach, enabling targeted therapies for EM patients based on their specific immunological and comorbidity profiles.

## 6 Conclusions and future directions

T cell biology is highly complex and involves a tightly regulated and multifaceted network of cell-cell interactions, signaling cascades, and developmental checkpoints that underpin immune function and homeostasis. T cells not only differentiate into a broad spectrum of specialized subsets including cytotoxic T cells, helper T cells (Th1, Th2, Th17, Tfh), and Tregs, but also exhibit immense diversity in their TCRs, enabling the recognition of a vast array of antigens. While most T cells require MHC-restricted antigen presentation, certain unconventional subsets such as γδ T cells and MAIT cells can function independent of classical MHC pathways. Moreover, memory T cells, including those specific to self-antigens, may persist and re-engage upon repeated exposures, adding another layer of complexity to immune surveillance and tolerance. These features of T cell biology become particularly relevant, and problematic in the context of EM. Ectopic EM lesions are autologous in origin, and yet they escape immune clearance, suggesting systemic and localized immune dysfunction. The lesion microenvironment is comprised of not only immune cells, but also epithelial-stromal-endothelial interactions tightly regulated under endocrine influence. In this ever-changing dynamic microenvironment, immune evasion is facilitated by both passive and active mechanisms. T cells likely become anergic, tolerogenic, or skewed toward anti-inflammatory phenotypes such as Th2. The Th2-dominant response, often associated with tissue remodeling and fibrosis, could be shaped by alarmins such as IL-33, which are secreted by epithelial and stromal cells in response to cellular stress or damage. Indeed, we and others have shown that IL-33 promote Th2 differentiation and facilitate the recruitment of ILC2s (56, 257), further amplifying the anti-inflammatory and tissue-supportive milieu.

Adding to this complexity is the fact that EM represents a unique form of sterile inflammation. Unlike pathogen induced immune responses, the chronic inflammation observed in EM arises in response to self-tissue likely deposited at ectopic sites via retrograde menstruation. Cells of the innate and adaptive immune system are recruited in response but remain functionally impaired, leading to the persistence of cellular debris. Chemokine gradients play a pivotal role in shaping immune cell infiltration. Specific subsets of chemokines attract corresponding immune cells, including effector T cells and regulatory populations. However, the presence of decoy receptors may modulate these gradients, acting as buffers that regulate the recruitment and localization of immune cells within the lesion microenvironment. This dynamic regulation of chemotaxis likely contributes to the establishment of an immune milieu that supports lesion persistence rather than clearance. Despite significant advances in characterizing individual immune cell populations involved in EM, a mechanistic understanding of how these cells interact, communicate, and influence lesion biology remains incomplete.

The advent of high-resolution techniques such as single-cell RNA sequencing, spatial transcriptomics, and advanced imaging modalities now offers unprecedented insight into the cellular composition, functional states, and intercellular communication within endometriotic lesions. These approaches will provide critical insights into the roles of different T cell subsets, including their activation status, effector functions, exhaustion phenotypes, and interactions with other immune and stromal cells. Ultimately, bridging these knowledge gaps is essential not only for elucidating the immunopathogenesis of EM but also for identifying targeted immunotherapies. This requires a systems-level understanding of immune-endocrine-resident cell interactions. Through such integrative, mechanistic studies, we should be able to develop precise, personalized interventions that address both the immunological and hormonal drivers of this highly heterogenous and complex disease affecting more than 200 million women globally (2).

## References

[1] Gołąbek-Grenda A Olejnik A . *In vitro* modeling of endometriosis and endometriotic microenvironment – Challenges and recent advances. Cell Signal. (2022) 97:110375. doi: 10.1016/j.cellsig.2022.110375, PMID: 35690293

[2] Bulun SE Yilmaz BD Sison C Miyazaki K Bernardi L Liu S . Endometriosis. Endocr Rev. (2019) 40:1048–79. doi: 10.1210/er.2018-00242, PMID: 30994890 PMC6693056

[3] Vallée A Feki A Ayoubi J-M . Endometriosis in transgender men: recognizing the missing pieces. Front Med. (2023) 10. doi: 10.3389/fmed.2023.1266131, PMID: 37720510 PMC10501128

[4] Broi MGD Ferriani RA Navarro PA . Ethiopathogenic mechanisms of endometriosis-related infertility. JBRA Assist Reprod. (2019) 23:273–80. doi: 10.5935/1518-0557.20190029, PMID: 31091056 PMC6724396

[5] Taylor HS Kotlyar AM Flores VA . Endometriosis is a chronic systemic disease: clinical challenges and novel innovations. Lancet. (2021) 397:839–52. doi: 10.1016/S0140-6736(21)00389-5, PMID: 33640070

[6] Ghai V Jan H Shakir F Haines P Kent A . Diagnostic delay for superficial and deep endometriosis in the United Kingdom. J Obstet Gynaecol (Lahore). (2020) 40:83–9. doi: 10.1080/01443615.2019.1603217, PMID: 31328629

[7] Horne AW Missmer SA . Pathophysiology, diagnosis, and management of endometriosis. BMJ. (2022) 379:e070750. doi: 10.1136/bmj-2022-070750, PMID: 36375827

[8] As-Sanie S Black R Giudice LC Valbrun TG Gupta J Jones B . Assessing research gaps and unmet needs in endometriosis. Am J Obstet Gynecol. (2019) 221:86–94. doi: 10.1016/j.ajog.2019.02.033, PMID: 30790565

[9] Capezzuoli T Orlandi G Clemenza S Ponziani I Sorbi F Vannuccini S . Gynaecologic and systemic comorbidities in patients with endometriosis: impact on quality of life and global health. Clin Exp Obstet Gynecol. (2022) 49:157. doi: 10.31083/j.ceog4907157, PMID: 41524059

[10] Lim HJ Sun J Min B Song M Kim TH Kim BJ . Endometriosis and adverse pregnancy outcomes: A nationwide population-based study. J Clin Med. (2023) 12:5392. doi: 10.3390/jcm12165392, PMID: 37629431 PMC10455587

[11] Zakhari A Delpero E McKeown S Tomlinson G Bougie O Murji A . Endometriosis reccurrence following post-operative hormonal suppression: a systematic review and meta-analysis. Hum Reprod Updat. (2021) 27:96–107. doi: 10.1093/humupd/dmaa033, PMID: 33020832 PMC7781224

[12] Soliman AM Surrey ES Bonafede M Nelson JK Vora JB Agarwal SK . Health care utilization and costs associated with endometriosis among women with medicaid insurance. J Manag Care Spec Pharm. (2019) 25:566–72. doi: 10.18553/jmcp.2019.25.5.566, PMID: 31039061 PMC10397603

[13] Darbà J Marsà A . Economic implications of endometriosis: A review. Pharmacoeconomics. (2022) 40:1143–58. doi: 10.1007/s40273-022-01211-0, PMID: 36344867

[14] Della Corte L Di Filippo C Gabrielli O Reppuccia S La Rosa VL Ragusa R . The burden of endometriosis on women’s lifespan: A narrative overview on quality of life and psychosocial wellbeing. Int J Environ Res Public Health. (2020) 17:4683. doi: 10.3390/ijerph17134683, PMID: 32610665 PMC7370081

[15] Simoens S Dunselman G Dirksen C Hummelshoj L Bokor A Brandes I . The burden of endometriosis: costs and quality of life of women with endometriosis and treated in referral centres. Hum Reprod. (2012) 27:1292–9. doi: 10.1093/humrep/des073, PMID: 22422778

[16] Le NXH Loret de Mola JR Bremer P Groesch K Wilson T Diaz-Sylvester P . Alteration of systemic and uterine endometrial immune populations in patients with endometriosis. Am J Reprod Immunol. (2021) 85:e13362. doi: 10.1111/aji.13362, PMID: 33070438

[17] Bouic PJ . Endometriosis and infertility: the hidden link between endometritis, hormonal imbalances and immune dysfunctions preventing implantation! JBRA Assist Reprod. (2023) 27:144–6. doi: 10.5935/1518-0557.20230015, PMID: 37348006 PMC10279451

[18] Miller JE Ahn SH Monsanto SP Khalaj K Koti M Tayade C . Implications of immune dysfunction on endometriosis associated infertility. Oncotarget. (2017) 8:7138–47. doi: 10.18632/oncotarget.12577, PMID: 27740937 PMC5351695

[19] Guo M Bafligil C Tapmeier T Hubbard C Manek S Shang C . Mass cytometry analysis reveals a distinct immune environment in peritoneal fluid in endometriosis: a characterisation study. BMC Med. (2020) 18:3. doi: 10.1186/s12916-019-1470-y, PMID: 31907005 PMC6945609

[20] Yuan M Li D An M Li Q Zhang L Wang G . Rediscovering peritoneal macrophages in a murine endometriosis model. Hum Reprod. (2017) 32:94–102. doi: 10.1093/humrep/dew274, PMID: 27816922

[21] Pizzo A Salmeri FM Ardita FV Sofo V Tripepi M Marsico S . Behaviour of cytokine levels in serum and peritoneal fluid of women with endometriosis. Gynecol Obstet Invest. (2002) 54:82–7. doi: 10.1159/000067717, PMID: 12566749

[22] Quanah J Ashjaei K Perricos A Kuessel L Husslein H Wenzl R . Endometriosis patients show an increased M2 response in the peritoneal CD14+low/CD68+low macrophage subpopulation coupled with an increase in the T-helper 2 and T-regulatory cells. Reprod Sci. (2020) 27:1920–31. doi: 10.1007/s43032-020-00211-9, PMID: 32572831 PMC7452931

[23] Mier-Cabrera J Jiménez-Zamudio L García-Latorre E Cruz-Orozco O Hernández-Guerrero C . Quantitative and qualitative peritoneal immune profiles, T-cell apoptosis and oxidative stress-associated characteristics in women with minimal and mild endometriosis. BJOG Int J Obstet Gynaecol. (2011) 118:6–16. doi: 10.1111/j.1471-0528.2010.02777.x, PMID: 21083865

[24] Podgaec S Abrao MS Dias JA Rizzo LV de Oliveira RM Baracat EC . Endometriosis: an inflammatory disease with a Th2 immune response component. Hum Reprod. (2007) 22:1373–9. doi: 10.1093/humrep/del516, PMID: 17234676

[25] Olkowska-Truchanowicz J Białoszewska A Zwierzchowska A Sztokfisz-Ignasiak A Janiuk I Dabrowski F . Peritoneal fluid from patients with ovarian endometriosis displays immunosuppressive potential and stimulates th2 response. Int J Mol Sci. (2021) 22:8134. doi: 10.3390/ijms22158134, PMID: 34360900 PMC8347337

[26] Santulli P Borghese B Chouzenoux S Vaiman D Borderie D Streuli I . Serum and peritoneal interleukin-33 levels are elevated in deeply infiltrating endometriosis. Hum Reprod. (2012) 27:2001–9. doi: 10.1093/humrep/des154, PMID: 22587998

[27] Kang Y-J Cho HJ Lee Y Park A Kim MJ Jeung IC . IL-17A and th17 cells contribute to endometrial cell survival by inhibiting apoptosis and NK cell mediated cytotoxicity of endometrial cells via ERK1/2 pathway. Immune Netw. (2023) 23:e14. doi: 10.4110/in.2023.23.e14, PMID: 37179747 PMC10166657

[28] Pashizeh F Mansouri R Davari-Tanha F Hosseini R Asgari Z Aghaei H . Alterations of CD4+T cell subsets in blood and peritoneal fluid in different stages of endometriosis. Int J Fertil Steril. (2020) 14:201–8. doi: 10.22074/ijfs.2020.6127, PMID: 33098386 PMC7604714

[29] Gogacz M Winkler I Bojarska-Junak A Tabarkiewicz J Semczuk A Rechberger T . Increased percentage of Th17 cells in peritoneal fluid is associated with severity of endometriosis. J Reprod Immunol. (2016) 117:39–44. doi: 10.1016/j.jri.2016.04.289, PMID: 27371900

[30] Khan KN Yamamoto K Fujishita A Muto H Koshiba A Kuroboshi H . Differential levels of regulatory T cells and T-helper-17 cells in women with early and advanced endometriosis. J Clin Endocrinol Metab. (2019) 104:4715–29. doi: 10.1210/jc.2019-00350, PMID: 31042291

[31] Jiang Y-P Peng Y-Q Wang L Qin J Zhang Y Zhao Y-Z . RNA-sequencing identifies differentially expressed genes in T helper 17 cells in peritoneal fluid of patients with endometriosis. J Reprod Immunol. (2022) 149:103453. doi: 10.1016/j.jri.2021.103453, PMID: 34839179

[32] Zhang X Xu H Lin J Qian Y Deng L . Peritoneal fluid concentrations of interleukin-17 correlate with the severity of endometriosis and infertility of this disorder. BJOG Int J Obstet Gynaecol. (2005) 112:1153–5. doi: 10.1111/j.1471-0528.2005.00639.x, PMID: 16045534

[33] Li Y Zhou Z Liang X Ding J He Y Sun S . Gut microbiota disorder contributes to the production of IL-17A that exerts chemotaxis via binding to IL-17RA in endometriosis. J Inflammation Res. (2024) 17:4199–217. doi: 10.2147/JIR.S458928, PMID: 38974001 PMC11225878

[34] Olkowska-Truchanowicz J Bocian K Maksym RB Białoszewska A Włodarczyk D Baranowski W . CD4+ CD25+ FOXP3+ regulatory T cells in peripheral blood and peritoneal fluid of patients with endometriosis. Hum Reprod. (2013) 28:119–24. doi: 10.1093/humrep/des346, PMID: 23019301

[35] Tanaka Y Mori T Ito F Koshiba A Takaoka O Hisashi K . Exacerbation of endometriosis due to regulatory T-cell dysfunction. J Clin Endocrinol Metab. (2017) 102:3206–17. doi: 10.1210/jc.2017-00052, PMID: 28575420

[36] Iwasaki K Makino T Maruyama T Matsubayashi H Nozawa S Yokokura T . Leukocyte subpopulations and natural killer activity in endometriosis. Int J Fertil Menopausal Stud. (1993) 38:229–34. 8401682

[37] Lessey BA Nagarkatti M Zhou J Young SL Adur MK Nowak RA . Peripheral and local cytokines including IL-8, IL-9 AND IL-17 are elevated in endometriosis: an *in vivo* and *in vitro* analysis before and after surgery suggests a mechanism for endometrial dysfunction. Fertil Steril. (2012) 98:S92. doi: 10.1016/j.fertnstert.2012.07.337, PMID: 41936479

[38] Tarumi Y Mori T Okimura H Maeda E Tanaka Y Kataoka H . Interleukin-9 produced by helper T cells stimulates interleukin-8 expression in endometriosis. Am J Reprod Immunol. (2021) 86. doi: 10.1111/aji.13380, PMID: 33210782

[39] Guo S Zhang Y Wang L Qiu W . Association of natural killer T cells with staging of endometriosis. Nan Fang Yi Ke Da Xue Xue Bao. (2012) 32:1322–4. 22985574

[40] Li C Lu Z Bi K Wang K Xu Y Guo P . CD4(+)/CD8(+) mucosa-associated invariant T cells foster the development of endometriosis: a pilot study. Reprod Biol Endocrinol. (2019) 17:78. doi: 10.1186/s12958-019-0524-5, PMID: 31615517 PMC6794756

[41] Takamura M Koga K Izumi G Hirata T Harada M Hirota Y . Simultaneous detection and evaluation of four subsets of CD4+ T lymphocyte in lesions and peripheral blood in endometriosis. Am J Reprod Immunol. (2015) 74:480–6. doi: 10.1111/aji.12426, PMID: 26387982

[42] Kidd P . Th1/Th2 balance: the hypothesis, its limitations, and implications for health and disease. Altern Med Rev. (2003) 8:223–46. 12946237

[43] Podgaec S Dias Junior JA Chapron C Oliveira RMD Baracat EC Abrão MS . Th1 and Th2 immune responses related to pelvic endometriosis. Rev Assoc Med Bras. (2010) 56:92–8. doi: 10.1590/S0104-42302010000100022, PMID: 20339793

[44] Sisnett DJ Zutautas KB Miller JE Lingegowda H Ahn SH McCallion A . The dysregulated IL-23/TH17 axis in endometriosis pathophysiology. J Immunol. (2024) 212:1428–41. doi: 10.4049/jimmunol.2400018, PMID: 38466035

[45] Ahn SH Edwards AK Singh SS Young SL Lessey BA Tayade C . IL-17A contributes to the pathogenesis of endometriosis by triggering proinflammatory cytokines and angiogenic growth factors. J Immunol. (2015) 195:2591–600. doi: 10.4049/jimmunol.1501138, PMID: 26259585 PMC4561197

[46] Delbandi A-A Mahmoudi M Shervin A Farhangnia P Mohammadi T Zarnani A-H . Increased circulating T helper 17 (TH17) cells and endometrial tissue IL-17-producing cells in patients with endometriosis compared with non-endometriotic subjects. Reprod Biol. (2025) 25:101019. doi: 10.1016/j.repbio.2025.101019, PMID: 40222069

[47] Le N Cregger M Fazleabas A Braundmeier-Fleming A . Effects of endometriosis on immunity and mucosal microbial community dynamics in female olive baboons. Sci Rep. (2022) 12:1590. doi: 10.1038/s41598-022-05499-y, PMID: 35102185 PMC8803974

[48] Hudecek R Kohlova B Siskova I Piskacek M Knight A . Blocking of ephA2 on endometrial tumor cells reduces susceptibility to vdelta1 gamma-delta T-cell-mediated killing. Front Immunol. (2021) 12:752646. doi: 10.3389/fimmu.2021.752646, PMID: 34691070 PMC8529280

[49] Correa FJS Andres MP Rocha TP Carvalho AEZ Aloia TPA Corpa MVN . Invariant Natural Killer T-cells and their subtypes may play a role in the pathogenesis of endometriosis. Clin (Sao Paulo). (2022) 77:100032. doi: 10.1016/j.clinsp.2022.100032, PMID: 35576870 PMC9118517

[50] Wu X-G Chen J-J Zhou H-L Wu Y Lin F Shi J . Identification and validation of the signatures of infiltrating immune cells in the eutopic endometrium endometria of women with endometriosis. Front Immunol. (2021) 12:671201. doi: 10.3389/fimmu.2021.671201, PMID: 34539624 PMC8446207

[51] Poli-Neto OB Carlos D Favaretto A Rosa-e-Silva JC Meola J Tiezzi D . Eutopic endometrium from women with endometriosis and chlamydial endometritis share immunological cell types and DNA repair imbalance: A transcriptome meta-analytical perspective. J Reprod Immunol. (2021) 145:103307. doi: 10.1016/j.jri.2021.103307, PMID: 33725527

[52] Antsiferova YS Sotnikova NY Posiseeva LV Shor AL . Changes in the T-helper cytokine profile and in lymphocyte activation at the systemic and local levels in women with endometriosis. Fertil Steril. (2005) 84:1705–11. doi: 10.1016/j.fertnstert.2005.05.066, PMID: 16359969

[53] Gueuvoghlanian-Silva BY Bellelis P Barbeiro DF Hernandes C Podgaec S . Treg and NK cells related cytokines are associated with deep rectosigmoid endometriosis and clinical symptoms related to the disease. J Reprod Immunol. (2018) 126:32–8. doi: 10.1016/j.jri.2018.02.003, PMID: 29477012

[54] Sadat Sandoghsaz R Montazeri F Shafienia H Mehdi Kalantar S Javaheri A Samadi M . Expression of miR-21 & IL-4 in endometriosis. Hum Immunol. (2024) 85:110746. doi: 10.1016/j.humimm.2023.110746, PMID: 38155071

[55] Aleksieva S Lingegowda H Sisnett DJ McCallion A Zutautas KB Vo DHN . Thymic stromal lymphopoietin contributes to endometriotic lesion proliferation and disease-associated inflammation. J Immunol. (2025) 214:399–412. doi: 10.1093/jimmun/vkae021, PMID: 40073108 PMC11952880

[56] Miller JE Monsanto SP Ahn SH Khalaj K Fazleabas AT Young SL . Interleukin-33 modulates inflammation in endometriosis. Sci Rep. (2017) 7:17903. doi: 10.1038/s41598-017-18224-x, PMID: 29263351 PMC5738435

[57] Xie C Lu C Liu Y Liu Z . Diagnostic gene biomarkers for predicting immune infiltration in endometriosis. BMC Womens Health. (2022) 22:184. doi: 10.1186/s12905-022-01765-3, PMID: 35585523 PMC9118874

[58] Tan Y Flynn WF Sivajothi S Luo D Bozal SB Davé M . Single-cell analysis of endometriosis reveals a coordinated transcriptional programme driving immunotolerance and angiogenesis across eutopic and ectopic tissues. Nat Cell Biol. (2022) 24:1306–18. doi: 10.1038/s41556-022-00961-5, PMID: 35864314 PMC9901845

[59] Zhong Q Yang F Chen X Li J Zhong C Chen S . Patterns of immune infiltration in endometriosis and their relationship to r-AFS stages. Front Genet. (2021) 12:631715. doi: 10.3389/fgene.2021.631715, PMID: 34220927 PMC8249861

[60] Ganewatta SP Berbic M Luscombe G Markham R Fraser IS . The characteristic expression of CD3+ T cells, CD8+ T cells and CD57+ NK cells in distinct zones of peritoneal endometriotic lesions. J Endometr. (2010) 2:189–96. doi: 10.1177/228402651000200403, PMID: 41930703

[61] Witz CA Montoya IA Dey TD Schenken RS . Characterization of lymphocyte subpopulations and T cell activation in endometriosis. Am J Reprod Immunol. (1994) 32:173–9. doi: 10.1111/j.1600-0897.1994.tb01110.x, PMID: 7880400

[62] Huang Z-X Lin D-C Zhang H-Y Yang M-J Chen J-H Ding X-Y . The dysfunction of CD8+ T cells triggered by endometriotic stromal cells promotes the immune survival of endometriosis. Immunology. (2024) 172:469–85. doi: 10.1111/imm.13786, PMID: 38544333

[63] Bunis DG Wang W Vallvé-Juanico J Houshdaran S Sen S Ben Soltane I . Whole-tissue deconvolution and scRNAseq analysis identify altered endometrial cellular compositions and functionality associated with endometriosis. Front Immunol. (2022) 12:788315. doi: 10.3389/fimmu.2021.788315, PMID: 35069565 PMC8766492

[64] Poli-Neto OB Meola J Rosa-e-Silva JC Tiezzi D . Transcriptome meta-analysis reveals differences of immune profile between eutopic endometrium from stage I-II and III-IV endometriosis independently of hormonal milieu. Sci Rep. (2020) 10:313. doi: 10.1038/s41598-019-57207-y, PMID: 31941945 PMC6962450

[65] Miller JE Lingegowda H Sisnett DJ Metz CN Gregersen PK Koti M . T helper 17 axis and endometrial macrophage disruption in menstrual effluent provides potential insights into the pathogenesis of endometriosis. F&S Sci. (2022) 3:279–87. doi: 10.1016/j.xfss.2022.04.007, PMID: 35697654

[66] Schmitz T Hoffmann V Olliges E Bobinger A Popovici R Nößner E . Reduced frequency of perforin-positive CD8+ T cells in menstrual effluent of endometriosis patients. J Reprod Immunol. (2021) 148:103424. doi: 10.1016/j.jri.2021.103424, PMID: 34563756

[67] Imam T Park S Kaplan MH Olson MR . Effector T helper cell subsets in inflammatory bowel diseases. Front Immunol. (2018) 9:1212. doi: 10.3389/fimmu.2018.01212, PMID: 29910812 PMC5992276

[68] Barbara G Cremon C Carini G Bellacosa L Zecchi L De Giorgio R . The immune system in irritable bowel syndrome. J Neurogastroenterol Motil. (2011) 17:349–59. doi: 10.5056/jnm.2011.17.4.349, PMID: 22148103 PMC3228974

[69] Lloyd CM Hessel EM . Functions of T cells in asthma: more than just T(H)2 cells. Nat Rev Immunol. (2010) 10:838–48. doi: 10.1038/nri2870, PMID: 21060320 PMC3807783

[70] Chin A Small A Wong SW Wechalekar MD . T cell dysregulation in rheumatoid arthritis: from genetic susceptibility to established disease. Curr Rheumatol Rep. (2025) 27:14. doi: 10.1007/s11926-025-01180-1, PMID: 39862300 PMC11762599

[71] Reis JL Rosa NN Martins C Ângelo-Dias M Borrego LM Lima J . The role of NK and T cells in endometriosis. Int J Mol Sci. (2024) 25:10141. doi: 10.3390/ijms251810141, PMID: 39337624 PMC11432446

[72] Yang H Zhuang Y . The deviations of CD4 + T cells during peripheral blood and peritoneal fluid of endometriosis: a systematic review and meta-analysis. Arch Gynecol Obstet. (2023) 308:1431–46. doi: 10.1007/s00404-023-06964-3, PMID: 36840769

[73] Blanco LP Salmeri N Temkin SM Shanmugam VK Stratton P . Endometriosis and autoimmunity. Autoimmun Rev. (2025) 24:103752. doi: 10.1016/j.autrev.2025.103752, PMID: 39828017 PMC12153059

[74] Chen S Chai X Wu X . Bioinformatical analysis of the key differentially expressed genes and associations with immune cell infiltration in development of endometriosis. BMC Genomic Data. (2022) 23:20. doi: 10.1186/s12863-022-01036-y, PMID: 35303800 PMC8932180

[75] Garmendia JV De Sanctis CV Hajdúch M De Sanctis JB . Endometriosis: an immunologist’s perspective. Int J Mol Sci. (2025) 26:5193. doi: 10.3390/ijms26115193, PMID: 40508002 PMC12154487

[76] Fan D Wang X Shi Z Jiang Y Zheng B Xu L . Understanding endometriosis from an immunomicroenvironmental perspective. Chin Med J (Engl). (2023) 136:1897–909. doi: 10.1097/CM9.0000000000002649, PMID: 37439327 PMC10431529

[77] Xia T Zeng K Peng Q Wu X Lei X . Clinical significance of serum Th1/Th2 cytokines in patients with endometriosis. Women Health. (2023) 63:73–82. doi: 10.1080/03630242.2022.2144986, PMID: 36581403

[78] Walker JA McKenzie ANJ . TH2 cell development and function. Nat Rev Immunol. (2018) 18:121–33. doi: 10.1038/nri.2017.118, PMID: 29082915

[79] Shih H-Y Sciumè G Poholek AC Vahedi G Hirahara K Villarino AV . Transcriptional and epigenetic networks of helper T and innate lymphoid cells. Immunol Rev. (2014) 261:23–49. doi: 10.1111/imr.12208, PMID: 25123275 PMC4321863

[80] Lin K-R Li P-X Zhu X-H Mao X-F Peng J-L Chen X-P . Peripheral immune characteristics and subset disorder in reproductive females with endometriosis. Front Immunol. (2024) 15:1431175. doi: 10.3389/fimmu.2024.1431175, PMID: 39669572 PMC11634862

[81] Morimoto Y Hirahara K Kiuchi M Wada T Ichikawa T Kanno T . Amphiregulin-producing pathogenic memory T helper 2 cells instruct eosinophils to secrete osteopontin and facilitate airway fibrosis. Immunity. (2018) 49:134–150.e6. doi: 10.1016/j.immuni.2018.04.023, PMID: 29958800

[82] Vissers G Giacomozzi M Verdurmen W Peek R Nap A . The role of fibrosis in endometriosis: a systematic review. Hum Reprod Update. (2024) 30:706–50. doi: 10.1093/humupd/dmae023, PMID: 39067455 PMC11532625

[83] Shafrir AL Palmor MC Fourquet J DiVasta AD Farland LV Vitonis AF . Co-occurrence of immune-mediated conditions and endometriosis among adolescents and adult women. Am J Reprod Immunol. (2021) 86. doi: 10.1111/aji.13404, PMID: 33583078 PMC8243788

[84] Shigesi N Kvaskoff M Kirtley S Feng Q Fang H Knight JC . The association between endometriosis and autoimmune diseases: a systematic review and meta-analysis. Hum Reprod Update. (2019) 25:486–503. doi: 10.1093/humupd/dmz014, PMID: 31260048 PMC6601386

[85] Kumar R Theiss AL Venuprasad K . RORγt protein modifications and IL-17-mediated inflammation. Trends Immunol. (2021) 42:1037–50. doi: 10.1016/j.it.2021.09.005, PMID: 34635393 PMC8556362

[86] Capone A Volpe E . Transcriptional regulators of T helper 17 cell differentiation in health and autoimmune diseases. Front Immunol. (2020) 11. doi: 10.3389/fimmu.2020.00348, PMID: 32226427 PMC7080699

[87] Maddur MS Miossec P Kaveri SV Bayry J . Th17 cells: biology, pathogenesis of autoimmune and inflammatory diseases, and therapeutic strategies. Am J Pathol. (2012) 181:8–18. doi: 10.1016/j.ajpath.2012.03.044, PMID: 22640807

[88] Stritesky GL Yeh N Kaplan MH . IL-23 promotes maintenance but not commitment to the Th17 lineage. J Immunol. (2008) 181:5948–55. doi: 10.4049/jimmunol.181.9.5948, PMID: 18941183 PMC2678905

[89] Langrish CL Chen Y Blumenschein WM Mattson J Basham B Sedgwick JD . IL-23 drives a pathogenic T cell population that induces autoimmune inflammation. J Exp Med. (2005) 201:233–40. doi: 10.1084/jem.20041257, PMID: 15657292 PMC2212798

[90] Shi G Cox CA Vistica BP Tan C Wawrousek EF Gery I . Phenotype switching by inflammation-inducing polarized th17 cells, but not by th1 cells. J Immunol. (2008) 181:7205–13. doi: 10.4049/jimmunol.181.10.7205, PMID: 18981142 PMC2665021

[91] Wu X Tian J Wang S . Insight into non-pathogenic th17 cells in autoimmune diseases. Front Immunol. (2018) 9:1112. doi: 10.3389/fimmu.2018.01112, PMID: 29892286 PMC5985293

[92] Hirota K Turner J-E Villa M Duarte JH Demengeot J Steinmetz OM . Plasticity of Th17 cells in Peyer’s patches is responsible for the induction of T cell-dependent IgA responses. Nat Immunol. (2013) 14:372–9. doi: 10.1038/ni.2552, PMID: 23475182 PMC3672955

[93] Gagliani N Amezcua Vesely MC Iseppon A Brockmann L Xu H Palm NW . Th17 cells transdifferentiate into regulatory T cells during resolution of inflammation. Nature. (2015) 523:221–5. doi: 10.1038/nature14452, PMID: 25924064 PMC4498984

[94] Omenetti S Pizarro TT . The treg/th17 axis: A dynamic balance regulated by the gut microbiome. Front Immunol. (2015) 6:639. doi: 10.3389/fimmu.2015.00639, PMID: 26734006 PMC4681807

[95] Lee GR . The balance of th17 versus treg cells in autoimmunity. Int J Mol Sci. (2018) 19:710–1. doi: 10.3390/ijms19030730, PMID: 29510522 PMC5877591

[96] Yan J-B Luo M-M Chen Z-Y He B-H . The function and role of the th17/treg cell balance in inflammatory bowel disease. J Immunol Res. (2020) 2020:8813558. doi: 10.1155/2020/8813558, PMID: 33381606 PMC7755495

[97] Stadhouders R Lubberts E Hendriks RW . A cellular and molecular view of T helper 17 cell plasticity in autoimmunity. J Autoimmun. (2018) 87:1–15. doi: 10.1016/j.jaut.2017.12.007, PMID: 29275836

[98] Zhang J Zhao W Zhou Y Xi S Xu X Du X . Pyroptotic T cell-derived active IL-16 has a driving function in ovarian endometriosis development. Cell Rep Med. (2024) 5:101476. doi: 10.1016/J.XCRM.2024.101476, PMID: 38508138 PMC10983113

[99] Miller JE Ahn SH Marks RM Monsanto SP Fazleabas AT Koti M . IL-17A modulates peritoneal macrophage recruitment and M2 polarization in endometriosis. Front Immunol. (2020) 11:108. doi: 10.3389/fimmu.2020.00108, PMID: 32117261 PMC7034338

[100] Ballek O Valečka J Dobešová M Broučková A Manning J Řehulka P . TCR triggering induces the formation of lck-RACK1-actinin-1 multiprotein network affecting lck redistribution. Front Immunol. (2016) 7:449. doi: 10.3389/fimmu.2016.00449, PMID: 27833610 PMC5081367

[101] Cheng Q Yang X Zou T Sun L Zhang X Deng L . RACK1 enhances STAT3 stability and promotes T follicular helper cell development and function during blood-stage Plasmodium infection in mice. PloS Pathog. (2024) 20:e1012352. doi: 10.1371/journal.ppat.1012352, PMID: 39024388 PMC11288429

[102] Chen S Lin F Shin ME Wang F Shen L Hamm HE . RACK1 regulates directional cell migration by acting on G betagamma at the interface with its effectors PLC beta and PI3K gamma. Mol Biol Cell. (2008) 19:3909–22. doi: 10.1091/mbc.e08-04-0433, PMID: 18596232 PMC2526680

[103] Qiu G Liu J Cheng Q Wang Q Jing Z Pei Y . Impaired autophagy and defective T cell homeostasis in mice with T cell-specific deletion of receptor for activated C kinase 1. Front Immunol. (2017) 8:575. doi: 10.3389/fimmu.2017.00575, PMID: 28572806 PMC5435764

[104] Zizolfi B Foreste V Gallo A Martone S Giampaolino P Di Spiezio Sardo A . Endometriosis and dysbiosis: State of art. Front Endocrinol (Lausanne). (2023) 14:1140774. doi: 10.3389/fendo.2023.1140774, PMID: 36891056 PMC9986482

[105] Crotty S . T follicular helper cell differentiation, function, and roles in disease. Immunity. (2014) 41:529–42. doi: 10.1016/j.immuni.2014.10.004, PMID: 25367570 PMC4223692

[106] Gutiérrez-Melo N Baumjohann D . T follicular helper cells in cancer. Trends Cancer. (2023) 9:309–25. doi: 10.1016/j.trecan.2022.12.007, PMID: 36642575

[107] Liu X Yan X Zhong B Nurieva RI Wang A Wang X . Bcl6 expression specifies the T follicular helper cell program. vivo J Exp Med. (2012) 209:1841–52. doi: 10.1084/jem.20120219, PMID: 22987803 PMC3457730

[108] Wei X Niu X . T follicular helper cells in autoimmune diseases. J Autoimmun. (2023) 134:102976. doi: 10.1016/j.jaut.2022.102976, PMID: 36525939

[109] Menzhinskaya IV Pavlovich SV Melkumyan AG Chuprynin VD Yarotskaya EL Sukhikh GT . Potential significance of serum autoantibodies to endometrial antigens, α-enolase and hormones in non-invasive diagnosis and pathogenesis of endometriosis. Int J Mol Sci. (2023) 24. doi: 10.3390/ijms242115578, PMID: 37958566 PMC10649774

[110] Zutautas KB Yolmo P Xu M Childs T Koti M Tayade C . Tertiary lymphoid structures in endometriosis. F&S Sci. (2024) 5:335–41. doi: 10.1016/j.xfss.2024.10.001, PMID: 39370108

[111] Zhu T Du Y Jin B Zhang F Guan Y . Identifying immune cell infiltration and hub genes related to M2 macrophages in endometriosis by bioinformatics analysis. Reprod Sci. (2023) 30:3388–99. doi: 10.1007/s43032-023-01227-7, PMID: 37308800

[112] Chen W Song J Huang W Han X Zhuang J Li S . Bioinformatics-based identification of platelet-related genes and analysis of immune infiltration landscape in endometriosis. Clin Lab. (2025) 71. doi: 10.7754/Clin.Lab.2025.241004, PMID: 40663090

[113] Bailey-Bucktrout SL Martinez-Llordella M Zhou X Anthony B Rosenthal W Luche H . Self-antigen-driven activation induces instability of regulatory T cells during an inflammatory autoimmune response. Immunity. (2013) 39:949–62. doi: 10.1016/j.immuni.2013.10.016, PMID: 24238343 PMC3912996

[114] Nussbaum L Chen YL Ogg GS . Role of regulatory T cells in psoriasis pathogenesis and treatment. Br J Dermatol. (2021) 184:14–24. doi: 10.1111/bjd.19380, PMID: 32628773

[115] Ohue Y Nishikawa H . Regulatory T (Treg) cells in cancer: Can Treg cells be a new therapeutic target? Cancer Sci. (2019) 110:2080–9. doi: 10.1111/cas.14069, PMID: 31102428 PMC6609813

[116] Knez J Kovačič B Goropevšek A . The role of regulatory T-cells in the development of endometriosis. Hum Reprod. (2024) 39:1367–80. doi: 10.1093/humrep/deae103, PMID: 38756099

[117] Hori S . FOXP3 as a master regulator of Treg cells. Nat Rev Immunol. (2021) 21:618–9. doi: 10.1038/s41577-021-00598-9, PMID: 34580451

[118] Goswami TK Singh M Dhawan M Mitra S Emran TB Rabaan AA . Regulatory T cells (Tregs) and their therapeutic potential against autoimmune disorders – Advances and challenges. Hum Vaccin Immunother. (2022) 18:2035117. doi: 10.1080/21645515.2022.2035117, PMID: 35240914 PMC9009914

[119] Zhou X Bailey-Bucktrout S Jeker LT Bluestone JA . Plasticity of CD4+ FoxP3+ T cells. Curr Opin Immunol. (2009) 21:281–5. doi: 10.1016/j.coi.2009.05.007, PMID: 19500966 PMC2733784

[120] Li M-Q Wang Y Chang K-K Meng Y-H Liu L-B Mei J . CD4+Foxp3+ regulatory T cell differentiation mediated by endometrial stromal cell-derived TECK promotes the growth and invasion of endometriotic lesions. Cell Death Dis. (2014) 5:e1436–6. doi: 10.1038/cddis.2014.414, PMID: 25275597 PMC4649519

[121] Hou X-X Wang X-Q Zhou W-J Li D-J . Regulatory T cells induce polarization of pro-repair macrophages by secreting sFGL2 into the endometriotic milieu. Commun Biol. (2021) 4:1–16. doi: 10.1038/s42003-021-02018-z, PMID: 33893391 PMC8065041

[122] Xiao F Liu X Guo S-W . Interleukin-33 derived from endometriotic lesions promotes fibrogenesis through inducing the production of profibrotic cytokines by regulatory T cells. Biomedicines. (2022) 10:2893. doi: 10.3390/biomedicines10112893, PMID: 36428461 PMC9687776

[123] Facciabene A Peng X Hagemann IS Balint K Barchetti A Wang L-P . Tumour hypoxia promotes tolerance and angiogenesis via CCL28 and Treg cells. Nature. (2011) 475:226–30. doi: 10.1038/nature10169, PMID: 21753853

[124] Woidacki K Meyer N Schumacher A Goldschmidt A Maurer M Zenclussen AC . Transfer of regulatory T cells into abortion-prone mice promotes the expansion of uterine mast cells and normalizes early pregnancy angiogenesis. Sci Rep. (2015) 5:13938. doi: 10.1038/srep13938, PMID: 26355667 PMC4565045

[125] Wang X-Q Zhou W-J Luo X-Z Tao Y Li D-J . Synergistic effect of regulatory T cells and proinflammatory cytokines in angiogenesis in the endometriotic milieu. Hum Reprod. (2017) 32:1304–17. doi: 10.1093/humrep/dex067, PMID: 28383711

[126] Mbarik M Kaabachi W Henidi B Sassi FH Hamzaoui K . Soluble ST2 and IL-33: Potential markers of endometriosis in the Tunisian population. Immunol Lett. (2015) 166:1–5. doi: 10.1016/j.imlet.2015.05.002, PMID: 25977120

[127] Kawai K Uchiyama M Hester J Issa F . IL-33 drives the production of mouse regulatory T cells with enhanced *in vivo* suppressive activity in skin transplantation. Am J Transplant. (2020) 21:978. doi: 10.1111/ajt.16266, PMID: 33314772 PMC7613121

[128] Schiering C Krausgruber T Chomka A Fröhlich A Adelmann K Wohlfert EA . The alarmin IL-33 promotes regulatory T-cell function in the intestine. Nature. (2014) 513:564–8. doi: 10.1038/nature13577, PMID: 25043027 PMC4339042

[129] Hatzioannou A Banos A Sakelaropoulos T Fedonidis C Vidali M-S Köhne M . An intrinsic role of IL-33 in Treg cell–mediated tumor immunoevasion. Nat Immunol. (2020) 21:75–85. doi: 10.1038/s41590-019-0555-2, PMID: 31844326 PMC7030950

[130] Mühl H Pfeilschifter J . Anti-inflammatory properties of pro-inflammatory interferon-γ. Int Immunopharmacol. (2003) 3:1247–55. doi: 10.1016/S1567-5769(03)00131-0, PMID: 12890422

[131] Zelová H Hošek J . TNF-α signalling and inflammation: interactions between old acquaintances. Inflammation Res. (2013) 62:641–51. doi: 10.1007/s00011-013-0633-0, PMID: 23685857

[132] Crispin JC Tsokos GC . Cancer immunosurveillance by CD8 T cells. F1000Research. (2020) 9:80. doi: 10.12688/f1000research.21150.1, PMID: 32089832 PMC7001753

[133] Carvalheiro H da Silva JAP Souto-Carneiro MM . Potential roles for CD8+ T cells in rheumatoid arthritis. Autoimmun Rev. (2013) 12:401–9. doi: 10.1016/j.autrev.2012.07.011, PMID: 22841983

[134] Hussein MR Fathi NA El-Din AME Hassan HI Abdullah F Al-Hakeem E . Alterations of the CD4+, CD8+ T cell subsets, interleukins-1β, IL-10, IL-17, tumor necrosis factor-α and soluble intercellular adhesion molecule-1 in rheumatoid arthritis and osteoarthritis: preliminary observations. Pathol Oncol Res. (2008) 14:321–8. doi: 10.1007/s12253-008-9016-1, PMID: 18392953

[135] Koh CH Lee S Kwak M Kim BS Chung Y . CD8 T-cell subsets: heterogeneity, functions, and therapeutic potential. Exp Mol Med. (2023) 55:2287–99. doi: 10.1038/s12276-023-01105-x, PMID: 37907738 PMC10689838

[136] Ciric B El-behi M Cabrera R Zhang G-X Rostami A . IL-23 drives pathogenic IL-17-producing CD8+ T cells. J Immunol. (2009) 182:5296–305. doi: 10.4049/jimmunol.0900036, PMID: 19380776

[137] Kisovar A Becker CM Granne I Southcombe JH . The role of CD8+ T cells in endometriosis: a systematic review. Front Immunol. (2023) 14:1225639. doi: 10.3389/fimmu.2023.1225639, PMID: 37497226 PMC10366819

[138] St. Paul M Ohashi PS . The roles of CD8+ T cell subsets in antitumor immunity. Trends Cell Biol. (2020) 30:695–704. doi: 10.1016/j.tcb.2020.06.003, PMID: 32624246

[139] Barnaba V . T cell memory in infection, cancer, and autoimmunity. Front Immunol. (2022) 12:811968. doi: 10.3389/fimmu.2021.811968, PMID: 35069600 PMC8771143

[140] Xing Y Hogquist KA . T-cell tolerance: central and peripheral. Cold Spring Harb Perspect Biol. (2012) 4:a006957–a006957. doi: 10.1101/cshperspect.a006957, PMID: 22661634 PMC3367546

[141] Wherry EJ Kurachi M . Molecular and cellular insights into T cell exhaustion. Nat Rev Immunol. (2015) 15:486–99. doi: 10.1038/nri3862, PMID: 26205583 PMC4889009

[142] Shin H Wherry EJ . CD8 T cell dysfunction during chronic viral infection. Curr Opin Immunol. (2007) 19:408–15. doi: 10.1016/j.coi.2007.06.004, PMID: 17656078

[143] Lamceva J Uljanovs R Strumfa I . The main theories on the pathogenesis of endometriosis. Int J Mol Sci. (2023) 24:4254. doi: 10.3390/ijms24054254, PMID: 36901685 PMC10001466

[144] Wu MH Yang BC Lee YC Wu PL Hsu CC . The differential expression of intercellular adhesion molecule-1 (ICAM-1) and regulation by interferon-gamma during the pathogenesis of endometriosis. Am J Reprod Immunol. (2004) 51:373–80. doi: 10.1111/j.1600-0897.2004.00171.x, PMID: 15212674

[145] Thomson AJ Greer MR Young A Boswell F Telfer JF Cameron IT . Expression of intercellular adhesion molecules ICAM-1 and ICAM-2 in human endometrium: regulation by interferon-γ. Mol Hum Reprod. (1999) 5:64–70. doi: 10.1093/molehr/5.1.64, PMID: 10050664

[146] De Placido G Alviggi C Di Palma G Carravetta C Matarese G Landino G . Serum concentrations of soluble human leukocyte class I antigens and of the soluble intercellular adhesion molecule-1 in endometriosis: relationship with stage and non-pigmented peritoneal lesions. Hum Reprod. (1998) 13:3206–10. doi: 10.1093/humrep/13.11.3206, PMID: 9853882

[147] Wu M-H Yang B-C Hsu C-C Lee Y-C Huang K-E . The expression of soluble intercellular adhesion molecule-1 in endometriosis. Fertil Steril. (1998) 70:1139–42. doi: 10.1016/S0015-0282(98)00384-7, PMID: 9848307

[148] Imperiale L Nisolle M Noël JC Fastrez M . Three types of endometriosis: pathogenesis, diagnosis and treatment. State Art J Clin Med. (2023) 12:994. doi: 10.3390/jcm12030994, PMID: 36769642 PMC9918005

[149] Liu M Li Y Yuan Y Jiang M Yin P Yang D . Peri-implantation treatment with TNF-α inhibitor for endometriosis and/or adenomyosis women undergoing frozen-thawed embryo transfer: A retrospective cohort study. J Reprod Immunol. (2025) 167. doi: 10.1016/j.jri.2024.104415, PMID: 39700679

[150] Rahmawati NY Ahsan F Santoso B Mufid AF Sa’adi A Dwiningsih SR . Role of TNF superfamily members lymphotoxin-α, sCD40L, and TNF-α in endometriosis-related infertility. J Endometr Pelvic Pain Disord. (2024) 16:79–88. doi: 10.1177/22840265241231722, PMID: 41930703

[151] Xu H Zou H Wen Q Xing X Xu N Wu S . Association between endometriosis and arthritis: results from NHANES 1999-2006, genetic correlation analysis, and Mendelian randomization study. Front Immunol. (2024) 15:1424648. doi: 10.3389/fimmu.2024.1424648, PMID: 39136014 PMC11317389

[152] Huang BS Chang WH Wang KC Huang N Guo CY Chou YJ . Endometriosis might be inversely associated with developing chronic kidney disease: A population-based cohort study in Taiwan. Int J Mol Sci. (2016) 17. doi: 10.3390/ijms17071079, PMID: 27399682 PMC4964455

[153] Sinaii N Cleary SD Ballweg ML Nieman LK Stratton P . High rates of autoimmune and endocrine disorders, fibromyalgia, chronic fatigue syndrome and atopic diseases among women with endometriosis: A survey analysis. Human Reproduction. (2002) 17:2715–24. doi: 10.1093/humrep/17.10.2715, PMID: 12351553

[154] Carvalheiro H Duarte C Silva-Cardoso S Da Silva JAP Souto-Carneiro MM . CD8+ T cell profiles in patients with rheumatoid arthritis and their relationship to disease activity. Arthritis Rheumatol. (2015) 67:363–71. doi: 10.1002/art.38941, PMID: 25370956

[155] Hassany AE Tharwat S Mansour M Enein AF . TNF-α in the serum and synovial fluid of patients with rheumatoid arthritis: correlation with sonographic parameters: a cross-sectional study. Egypt Rheumatol Rehabil. (2024) 51:22. doi: 10.1186/s43166-024-00256-7, PMID: 41933398

[156] Tsuda M Hamade H Thomas LS Salumbides BC Potdar AA Wong MH . A role for BATF3 in TH9 differentiation and T-cell-driven mucosal pathologies. Mucosal Immunol. (2019) 12:644–55. doi: 10.1038/s41385-018-0122-4, PMID: 30617301 PMC6462229

[157] Veldhoen M Uyttenhove C van Snick J Helmby H Westendorf A Buer J . Transforming growth factor-β “reprograms” the differentiation of T helper 2 cells and promotes an interleukin 9–producing subset. Nat Immunol. (2008) 9:1341–6. doi: 10.1038/ni.1659, PMID: 18931678

[158] Micossé C von Meyenn L Steck O Kipfer E Adam C Simillion C . Human “Th9” cells are a subpopulation of PPAR-γ+ Th2 cells. Sci Immunol. (2019) 4. doi: 10.1126/sciimmunol.aat5943, PMID: 30658968

[159] Angkasekwinai P . Th9 cells in allergic disease. Curr Allergy Asthma Rep. (2019) 19:29. doi: 10.1007/s11882-019-0860-8, PMID: 30915580

[160] Monsanto SP Edwards AK Zhou J Nagarkatti P Nagarkatti M Young SL . Surgical removal of endometriotic lesions alters local and systemic proinflammatory cytokines in endometriosis patients. Fertil Steril. (2016) 105:968–977.e5. doi: 10.1016/j.fertnstert.2015.11.047, PMID: 26698677 PMC4851862

[161] McCallion A Nasirzadeh Y Lingegowda H Miller JE Khalaj K Ahn SH . Estrogen mediates inflammatory role of mast cells in endometriosis pathophysiology. Front Immunol. (2022) 13:961599. doi: 10.3389/fimmu.2022.961599, PMID: 36016927 PMC9396281

[162] McCallion A Zutautas KB Sisnett DJ Yolmo P Lingegowda H Ravishanker AK . Contributions of T helper 9 cells in endometriosis-associated inflammation and lesion growth. BioRxiv. (2025). doi: 10.1101/2025.04.04.647212, PMID: 41887800

[163] Maeda N Izumiya C Kusum T Masumoto T Yamashita C Yamamoto Y . Killer inhibitory receptor CD158a overexpression among natural killer cells in women with endometriosis is undiminished by laparoscopic surgery and gonadotropin releasing hormone agonist treatment. Am J Reprod Immunol. (2004) 51:364–72. doi: 10.1111/j.1600-0897.2004.00170.x, PMID: 15212673

[164] Sallusto F Geginat J Lanzavecchia A . Central memory and effector memory T cell subsets: function, generation, and maintenance. Annu Rev Immunol. (2004) 22:745–63. doi: 10.1146/annurev.immunol.22.012703.104702, PMID: 15032595

[165] Gebhardt T Wakim LM Eidsmo L Reading PC Heath WR Carbone FR . Memory T cells in nonlymphoid tissue that provide enhanced local immunity during infection with herpes simplex virus. Nat Immunol. (2009) 10:524–30. doi: 10.1038/ni.1718, PMID: 19305395

[166] Arbones ML Ord DC Ley K Ratech H Maynard-Curry C Otten G . Lymphocyte homing and leukocyte rolling and migration are impaired in L-selectin-deficient mice. Immunity. (1994) 1:247–60. doi: 10.1016/1074-7613(94)90076-0, PMID: 7534203

[167] Forster R Schubel A Breitfeld D Kremmer E Renner-Muller I Wolf E . CCR7 coordinates the primary immune response by establishing functional microenvironments in secondary lymphoid organs. Cell. (1999) 99:23–33. doi: 10.1016/s0092-8674(00)80059-8, PMID: 10520991

[168] Mackay LK Rahimpour A Ma JZ Collins N Stock AT Hafon ML . The developmental pathway for CD103(+)CD8+ tissue-resident memory T cells of skin. Nat Immunol. (2013) 14:1294–301. doi: 10.1038/ni.2744, PMID: 24162776

[169] Richter MV Topham DJ . The alpha1beta1 integrin and TNF receptor II protect airway CD8+ effector T cells from apoptosis during influenza infection. J Immunol. (2007) 179:5054–63. doi: 10.4049/jimmunol.179.8.5054, PMID: 17911590

[170] Mackay LK Stock AT Ma JZ Jones CM Kent SJ Mueller SN . Long-lived epithelial immunity by tissue-resident memory T (TRM) cells in the absence of persisting local antigen presentation. Proc Natl Acad Sci U S A. (2012) 109:7037–42. doi: 10.1073/pnas.1202288109, PMID: 22509047 PMC3344960

[171] Sallusto F Lenig D Forster R Lipp M Lanzavecchia A . Two subsets of memory T lymphocytes with distinct homing potentials and effector functions. Nature. (1999) 401:708–12. doi: 10.1038/44385, PMID: 10537110

[172] Wherry EJ Teichgraber V Becker TC Masopust D Kaech SM Antia R . Lineage relationship and protective immunity of memory CD8 T cell subsets. Nat Immunol. (2003) 4:225–34. doi: 10.1038/ni889, PMID: 12563257

[173] Schenkel JM Fraser KA Beura LK Pauken KE Vezys V Masopust D . T cell memory. Resident memory CD8 T cells trigger protective innate and adaptive immune responses. Science. (2014) 346:98–101. doi: 10.1126/science.1254536, PMID: 25170049 PMC4449618

[174] Ariotti S Beltman JB Chodaczek G Hoekstra ME van Beek AE Gomez-Eerland R . Tissue-resident memory CD8+ T cells continuously patrol skin epithelia to quickly recognize local antigen. Proc Natl Acad Sci U S A. (2012) 109:19739–44. doi: 10.1073/pnas.1208927109, PMID: 23150545 PMC3511734

[175] Sheridan BS Lefrancois L . Regional and mucosal memory T cells. Nat Immunol. (2011) 12:485–91. doi: 10.1038/ni.2029, PMID: 21739671 PMC3224372

[176] Fernandez-Ruiz D Ng WY Holz LE Ma JZ Zaid A Wong YC . Liver-resident memory CD8(+) T cells form a front-line defense against malaria liver-stage infection. Immunity. (2019) 51:780. doi: 10.1016/j.immuni.2019.09.019, PMID: 31618655

[177] Steinbach K Vincenti I Kreutzfeldt M Page N Muschaweckh A Wagner I . Brain-resident memory T cells represent an autonomous cytotoxic barrier to viral infection. J Exp Med. (2016) 213:1571–87. doi: 10.1084/jem.20151916, PMID: 27377586 PMC4986533

[178] Liu Z-Q Lu M-Y Liu B . Circulating CD56+ NKG2D+ NK cells and postoperative fertility in ovarian endometrioma. Sci Rep. (2020) 10:18598. doi: 10.1038/s41598-020-75570-z, PMID: 33122818 PMC7596045

[179] Hu Y Hu Q Li Y Lu L Xiang Z Yin Z . γδ T cells: origin and fate, subsets, diseases and immunotherapy. Signal Transduct Target Ther. (2023) 8:434. doi: 10.1038/s41392-023-01653-8, PMID: 37989744 PMC10663641

[180] Silva-Santos B Serre K Norell H . gammadelta T cells in cancer. Nat Rev Immunol. (2015) 15:683–91. doi: 10.1038/nri3904, PMID: 26449179

[181] Vantourout P Hayday A . Six-of-the-best: unique contributions of gammadelta T cells to immunology. Nat Rev Immunol. (2013) 13:88–100. doi: 10.1038/nri3384, PMID: 23348415 PMC3951794

[182] Foyle KL Robertson SA . Gamma delta (gammadelta) T cells in the female reproductive tract: active participants or indifferent bystanders in reproductive success? Discov Immunol. (2024) 3:kyae004. doi: 10.1093/discim/kyae004, PMID: 38863792 PMC11165432

[183] Heilig JS Tonegawa S . Diversity of murine gamma genes and expression in fetal and adult T lymphocytes. Nature. (1986) 322:836–40. doi: 10.1038/322836a0, PMID: 2943999

[184] Kashani E Föhse L Raha S Sandrock I Oberdörfer L Koenecke C . A clonotypic Vγ4Jγ1/Vδ5Dδ2Jδ1 innate γδ T-cell population restricted to the CCR6+CD27– subset. Nat Commun. (2015) 6:6477. doi: 10.1038/ncomms7477, PMID: 25765849

[185] Vantourout P Laing A Woodward MJ Zlatareva I Apolonia L Jones AW . Heteromeric interactions regulate butyrophilin (BTN) and BTN-like molecules governing γδ T cell biology. Proc Natl Acad Sci. (2018) 115:1039–44. doi: 10.1073/pnas.1701237115, PMID: 29339503 PMC5798315

[186] Kang S Wu Q Huang J Yang B Liang C Chi P . Tissue resident memory gammadeltaT cells in murine uterus expressed high levels of IL-17 promoting the invasion of trophocytes. Front Immunol. (2020) 11:588227. doi: 10.3389/fimmu.2020.588227, PMID: 33519808 PMC7840782

[187] Cai D Tang Y Yao X . Changes of gammadeltaT cell subtypes during pregnancy and their influences in spontaneous abortion. J Reprod Immunol. (2019) 131:57–62. doi: 10.1016/j.jri.2019.01.003, PMID: 30710888

[188] Monin L Ushakov DS Arnesen H Bah N Jandke A Munoz-Ruiz M . gammadelta T cells compose a developmentally regulated intrauterine population and protect against vaginal candidiasis. Mucosal Immunol. (2020) 13:969–81. doi: 10.1038/s41385-020-0305-7, PMID: 32472066 PMC7567646

[189] Kang S Wu Q Yang B Wu C . Estrogen enhanced the expression of IL-17 by tissue-resident memory gammadeltaT cells from uterus via interferon regulatory factor 4. FASEB J. (2022) 36:e22166. doi: 10.1096/fj.202101443RR, PMID: 35064703

[190] Liang Q Tong L Xiang L Shen S Pan C Liu C . Correlations of the expression of gammadelta T cells and their co-stimulatory molecules TIGIT, PD-1, ICOS and BTLA with PR and PIBF in the peripheral blood and decidual tissues of women with unexplained recurrent spontaneous abortion. Clin Exp Immunol. (2021) 203:55–65. doi: 10.1111/cei.13534, PMID: 33017473 PMC7744496

[191] Foord E Arruda LCM Gaballa A Klynning C Uhlin M . Characterization of ascites- and tumor-infiltrating gammadelta T cells reveals distinct repertoires and a beneficial role in ovarian cancer. Sci Transl Med. (2021) 13. doi: 10.1126/scitranslmed.abb0192, PMID: 33472952

[192] Gumperz JE Miyake S Yamamura T Brenner MB . Functionally distinct subsets of CD1d-restricted natural killer T cells revealed by CD1d tetramer staining. J Exp Med. (2002) 195:625–36. doi: 10.1084/jem.20011786, PMID: 11877485 PMC2193772

[193] Coquet JM Chakravarti S Kyparissoudis K McNab FW Pitt LA McKenzie BS . Diverse cytokine production by NKT cell subsets and identification of an IL-17-producing CD4-NK1.1- NKT cell population. Proc Natl Acad Sci U S A. (2008) 105:11287–92. doi: 10.1073/pnas.0801631105, PMID: 18685112 PMC2516267

[194] Brennan PJ Brigl M Brenner MB . Invariant natural killer T cells: an innate activation scheme linked to diverse effector functions. Nat Rev Immunol. (2013) 13:101–17. doi: 10.1038/nri3369, PMID: 23334244

[195] Lee PT Benlagha K Teyton L Bendelac A . Distinct functional lineages of human V(alpha)24 natural killer T cells. J Exp Med. (2002) 195:637–41. doi: 10.1084/jem.20011908, PMID: 11877486 PMC2193771

[196] Godfrey DI Rossjohn J . New ways to turn on NKT cells. J Exp Med. (2011) 208:1121–5. doi: 10.1084/jem.20110983, PMID: 21646400 PMC3173239

[197] Matsuda JL Mallevaey T Scott-Browne J Gapin L . CD1d-restricted iNKT cells, the “Swiss-Army knife” of the immune system. Curr Opin Immunol. (2008) 20:358–68. doi: 10.1016/j.coi.2008.03.018, PMID: 18501573 PMC2546701

[198] Michel ML Keller AC Paget C Fujio M Trottein F Savage PB . Identification of an IL-17-producing NK1.1(neg) iNKT cell population involved in airway neutrophilia. J Exp Med. (2007) 204:995–1001. doi: 10.1084/jem.20061551, PMID: 17470641 PMC2118594

[199] Bulmer JN Williams PJ Lash GE . Immune cells in the placental bed. Int J Dev Biol. (2010) 54:281–94. doi: 10.1387/ijdb.082763jb, PMID: 19876837

[200] Bendelac A Savage PB Teyton L . The biology of NKT cells. Annu Rev Immunol. (2007) 25:297–336. doi: 10.1146/annurev.immunol.25.022106.141711, PMID: 17150027

[201] Boyson JE Nagarkatti N Nizam L Exley MA Strominger JL . Gestation stage-dependent mechanisms of invariant natural killer T cell-mediated pregnancy loss. Proc Natl Acad Sci U S A. (2006) 103:4580–5. doi: 10.1073/pnas.0511025103, PMID: 16537414 PMC1450214

[202] van den Heuvel MJ Peralta CG Hatta K Han VK Clark DA . Decline in number of elevated blood CD3(+) CD56(+) NKT cells in response to intravenous immunoglobulin treatment correlates with successful pregnancy. Am J Reprod Immunol. (2007) 58:447–59. doi: 10.1111/j.1600-0897.2007.00529.x, PMID: 17922698

[203] Miko E Manfai Z Meggyes M Barakonyi A Wilhelm F Varnagy A . Possible role of natural killer and natural killer T-like cells in implantation failure after IVF. Reprod BioMed Online. (2010) 21:750–6. doi: 10.1016/j.rbmo.2010.07.012, PMID: 21051289

[204] Crispin JC Tsokos GC . Human TCR-alpha beta+ CD4- CD8- T cells can derive from CD8+ T cells and display an inflammatory effector phenotype. J Immunol. (2009) 183:4675–81. doi: 10.4049/jimmunol.0901533, PMID: 19734235 PMC2878279

[205] Rodriguez-Rodriguez N Apostolidis SA Penaloza-MacMaster P Martin Villa JM Barouch DH Tsokos GC . Programmed cell death 1 and Helios distinguish TCR-alphabeta+ double-negative (CD4-CD8-) T cells that derive from self-reactive CD8 T cells. J Immunol. (2015) 194:4207–14. doi: 10.4049/jimmunol.1402775, PMID: 25825451 PMC4503929

[206] Li H Adamopoulos IE Moulton VR Stillman IE Herbert Z Moon JJ . Systemic lupus erythematosus favors the generation of IL-17 producing double negative T cells. Nat Commun. (2020) 11:2859. doi: 10.1038/s41467-020-16636-4, PMID: 32503973 PMC7275084

[207] Juvet SC Zhang L . Double negative regulatory T cells in transplantation and autoimmunity: recent progress and future directions. J Mol Cell Biol. (2012) 4:48–58. doi: 10.1093/jmcb/mjr043, PMID: 22294241 PMC3269300

[208] Brandt D Hedrich CM . TCRalphabeta(+)CD3(+)CD4(-)CD8(-) (double negative) T cells in autoimmunity. Autoimmun Rev. (2018) 17:422–30. doi: 10.1016/j.autrev.2018.02.001, PMID: 29428806

[209] Alunno A Bistoni O Bartoloni E Caterbi S Bigerna B Tabarrini A . IL-17-producing CD4-CD8- T cells are expanded in the peripheral blood, infiltrate salivary glands and are resistant to corticosteroids in patients with primary Sjogren’s syndrome. Ann Rheum Dis. (2013) 72:286–92. doi: 10.1136/annrheumdis-2012-201511, PMID: 22904262

[210] Fischer K Voelkl S Heymann J Przybylski GK Mondal K Laumer M . Isolation and characterization of human antigen-specific TCR alpha beta+ CD4-CD8- double-negative regulatory T cells. Blood. (2005) 105:2828–35. doi: 10.1182/blood-2004-07-2583, PMID: 15572590

[211] Di Blasi D Boldanova T Mori L Terracciano L Heim MH De Libero G . Unique T-cell populations define immune-inflamed hepatocellular carcinoma. Cell Mol Gastroenterol Hepatol. (2020) 9:195–218. doi: 10.1016/j.jcmgh.2019.08.004, PMID: 31445190 PMC6957799

[212] Liu Z Meng Q Bartek J Jr Poiret T Persson O Rane L . Tumor-infiltrating lymphocytes (TILs) from patients with glioma. Oncoimmunology. (2017) 6:e1252894. doi: 10.1080/2162402X.2016.1252894, PMID: 28344863 PMC5353900

[213] Hall M Liu H Malafa M Centeno B Hodul PJ Pimiento J . Expansion of tumor-infiltrating lymphocytes (TIL) from human pancreatic tumors. J Immunother Cancer. (2016) 4:61. doi: 10.1186/s40425-016-0164-7, PMID: 27777771 PMC5067894

[214] Fang L Ly D Wang SS Lee JB Kang H Xu H . Targeting late-stage non-small cell lung cancer with a combination of DNT cellular therapy and PD-1 checkpoint blockade. J Exp Clin Cancer Res. (2019) 38:123. doi: 10.1186/s13046-019-1126-y, PMID: 30857561 PMC6413451

[215] Prins RM Incardona F Lau R Lee P Claus S Zhang W . Characterization of defective CD4-CD8- T cells in murine tumors generated independent of antigen specificity. J Immunol. (2004) 172:1602–11. doi: 10.4049/jimmunol.172.3.1602, PMID: 14734741

[216] Wu Z Zheng Y Sheng J Han Y Yang Y Pan H . CD3+CD4-CD8- (Double-negative) T cells in inflammation, immune disorders and cancer. Front Immunol. (2022) 13:816005. doi: 10.3389/fimmu.2022.816005, PMID: 35222392 PMC8866817

[217] Li H Tsokos GC . Double-negative T cells in autoimmune diseases. Curr Opin Rheumatol. (2021) 33:163–72. doi: 10.1097/BOR.0000000000000778, PMID: 33394752 PMC8018563

[218] Toubal A Nel I Lotersztajn S Lehuen A . Mucosal-associated invariant T cells and disease. Nat Rev Immunol. (2019) 19:643–57. doi: 10.1038/s41577-019-0191-y, PMID: 31308521

[219] Howson LJ Napolitani G Shepherd D Ghadbane H Kurupati P Preciado-Llanes L . MAIT cell clonal expansion and TCR repertoire shaping in human volunteers challenged with Salmonella Paratyphi A. Nat Commun. (2018) 9:253. doi: 10.1038/s41467-017-02540-x, PMID: 29343684 PMC5772558

[220] Tilloy F Treiner E Park SH Garcia C Lemonnier F de la Salle H . An invariant T cell receptor alpha chain defines a novel TAP-independent major histocompatibility complex class Ib-restricted alpha/beta T cell subpopulation in mammals. J Exp Med. (1999) 189:1907–21. doi: 10.1084/jem.189.12.1907, PMID: 10377186 PMC2192962

[221] Loh L Wang Z Sant S Koutsakos M Jegaskanda S Corbett AJ . Human mucosal-associated invariant T cells contribute to antiviral influenza immunity via IL-18-dependent activation. Proc Natl Acad Sci U S A. (2016) 113:10133–8. doi: 10.1073/pnas.1610750113, PMID: 27543331 PMC5018778

[222] van Wilgenburg B Scherwitzl I Hutchinson EC Leng T Kurioka A Kulicke C . MAIT cells are activated during human viral infections. Nat Commun. (2016) 7:11653. doi: 10.1038/ncomms11653, PMID: 27337592 PMC4931007

[223] Miyazaki Y Miyake S Chiba A Lantz O Yamamura T . Mucosal-associated invariant T cells regulate Th1 response in multiple sclerosis. Int Immunol. (2011) 23:529–35. doi: 10.1093/intimm/dxr047, PMID: 21712423

[224] Ju JK Cho Y-N Park K-J Kwak HD Jin HM Park SY . Activation, deficiency, and reduced IFN-γ Production of mucosal-associated invariant T cells in patients with inflammatory bowel disease. J Innate Immun. (2020) 12:422–34. doi: 10.1159/000507931, PMID: 32535589 PMC7506267

[225] Koppejan H Jansen DTSL Hameetman M Thomas R Toes REM van Gaalen FA . Altered composition and phenotype of mucosal-associated invariant T cells in early untreated rheumatoid arthritis. Arthritis Res Ther. (2019) 21:3. doi: 10.1186/s13075-018-1799-1, PMID: 30611306 PMC6321723

[226] Sugimoto C Konno T Wakao R Fujita H Fujita H Wakao H . Mucosal-associated invariant T cell is a potential marker to distinguish fibromyalgia syndrome from arthritis. PloS One. (2015) 10:e0121124. doi: 10.1371/journal.pone.0121124, PMID: 25853812 PMC4390316

[227] Ayaz L Celik SK Cayan F Aras-Ates N Tamer L . Functional association of interleukin-18 gene -607 C/A promoter polymorphisms with endometriosis. Fertil Steril. (2011) 95:298–300. doi: 10.1016/j.fertnstert.2010.07.1046, PMID: 20797704

[228] Kim J-Y Lee D-H Joo J-K Jin J-O Wang J-W Hong Y-S . Effects of peritoneal fluid from endometriosis patients on interferon-γ-induced protein-10 (CXCL10) and interleukin-8 (CXCL8) released by neutrophils and CD4 + T cells. Am J Reprod Immunol. (2009) 62:128–38. doi: 10.1111/j.1600-0897.2009.00722.x, PMID: 19694638

[229] Campanella GSV Medoff BD Manice LA Colvin RA Luster AD . Development of a novel chemokine-mediated. Vivo T Cell recruitment assay J Immunol Methods. (2008) 331:127–39. doi: 10.1016/j.jim.2007.12.002, PMID: 18206159 PMC2277098

[230] Nie CQ Bernard NJ Norman MU Amante FH Lundie RJ Crabb BS . IP-10-mediated T cell homing promotes cerebral inflammation over splenic immunity to malaria infection. Riley EM Ed PloS Pathog. (2009) 5:e1000369. doi: 10.1371/journal.ppat.1000369, PMID: 19343215 PMC2658824

[231] Manabe S Iwase A Goto M Kobayashi H Takikawa S Nagatomo Y . Expression and localization of CXCL16 and CXCR6 in ovarian endometriotic tissues. Arch Gynecol Obstet. (2011) 284:1567–72. doi: 10.1007/s00404-011-2002-y, PMID: 21773780

[232] Günther C Carballido-Perrig N Kaesler S Carballido JM Biedermann T . CXCL16 and CXCR6 are upregulated in psoriasis and mediate cutaneous recruitment of human CD8^+^ T cells. J Invest Dermatol. (2012) 132:626–34. doi: 10.1038/jid.2011.371, PMID: 22113484

[233] Nanki T Shimaoka T Hayashida K Taniguchi K Yonehara S Miyasaka N . Pathogenic role of the CXCL16–CXCR6 pathway in rheumatoid arthritis. Arthritis Rheumatol. (2005) 52:3004–14. doi: 10.1002/art.21301, PMID: 16200580

[234] Nishimoto-Kakiuchi A Sato I Nakano K Ohmori H Kayukawa Y Tanimura H . A long-acting anti–IL-8 antibody improves inflammation and fibrosis in endometriosis. Sci Transl Med. (2023) 15. doi: 10.1126/scitranslmed.abq5858, PMID: 36812343

[235] Butcher MJ Wu C-I Waseem T Galkina EV . CXCR6 regulates the recruitment of pro-inflammatory IL-17A-producing T cells into atherosclerotic aortas. Int Immunol. (2016) 28:255–61. doi: 10.1093/intimm/dxv068, PMID: 26614640 PMC4888347

[236] Hirata T Osuga Y Takamura M Kodama A Hirota Y Koga K . Recruitment of CCR6-expressing th17 cells by CCL 20 secreted from IL-1β-, TNF-α-, and IL-17A-stimulated endometriotic stromal cells. Endocrinology. (2010) 151:5468–76. doi: 10.1210/en.2010-0398, PMID: 20881253

[237] Olkowska-Truchanowicz J Sztokfisz-Ignasiak A Zwierzchowska A Janiuk I Dąbrowski F Korczak-Kowalska G . Endometriotic peritoneal fluid stimulates recruitment of CD4+CD25highFOXP3+ Treg cells. J Clin Med. (2021) 10:3789. doi: 10.3390/jcm10173789, PMID: 34501240 PMC8432020

[238] Wyatt J Fernando SM Powell SG Hill CJ Arshad I Probert C . The role of iron in the pathogenesis of endometriosis: a systematic review. Hum Reprod Open. (2023) 2023:hoad033. doi: 10.1093/hropen/hoad033, PMID: 37638130 PMC10457727

[239] Park W Song G Lim W Park S . Therapeutic effects of S-allyl-L-cysteine in a mouse endometriosis model and its immunomodulatory effects via regulation of T cell subsets and cytokine expression. Pharmacol Rep. (2024) 76:1089–99. doi: 10.1007/s43440-024-00625-1, PMID: 39093549

[240] Han Y Liu D Li L . PD-1/PD-L1 pathway: current researches in cancer. Am J Cancer Res. (2020) 10:727–42. PMC7136921 32266087

[241] Sobstyl M Mertowska P Mertowski S Zaborek-Łyczba M Dudziński D Polak G . The PD-1/PD-L1 gateway: peripheral immune regulation in the pathogenesis of endometriosis. Int J Mol Sci. (2024) 25. doi: 10.3390/ijms25126775, PMID: 38928479 PMC11203925

[242] Abramiuk M Bębnowska D Hrynkiewicz R Polak PN-R Kotarski J Roliński J . CLTA-4 expression is associated with the maintenance of chronic inflammation in endometriosis and infertility. Cells. (2021) 10. doi: 10.3390/cells10030487, PMID: 33668701 PMC7996292

[243] Tian W Liu M Liu Y Lv Q Cheng H Gu Y . TIM-3 regulates the proliferation by BDNF-mediated PI3K/AKT axis in the process of endometriosis. Mol Med. (2023) 29:170. doi: 10.1186/s10020-023-00768-6, PMID: 38114892 PMC10731854

[244] Li Y-Y Lin Y-K Li Y Liu X-H Li D-J Wang X-L . SCM-198 alleviates endometriosis by suppressing estrogen-ERα mediated differentiation and function of CD4+CD25+ Regulatory T cells. Int J Biol Sci. (2022) 18:1961–73. doi: 10.7150/ijbs.68224, PMID: 35342349 PMC8935231

[245] Maeda E Okimura H Tanaka Y Fujii M Tarumi Y Kataoka H . Adoptive transfer of regulatory T cells inhibits the progression of endometriosis-like lesions in regulatory T-cell-depleted mice. Hum Reprod. (2025) 40:926–37. doi: 10.1093/humrep/deaf054, PMID: 40180333

[246] Bunte K Beikler T . Th17 cells and the IL-23/IL-17 axis in the pathogenesis of periodontitis and immune-mediated inflammatory diseases. Int J Mol Sci. (2019) 20. doi: 10.3390/ijms20143394, PMID: 31295952 PMC6679067

[247] Gerenova J Manolova I Stanilova S . SERUM LEVELS OF INTERLEUKIN - 23 AND INTERLEUKIN - 17 IN HASHIMOTO’S THYROIDITIS. Acta Endocrinol (Bucharest Rom 2005). (2019) 5:74–9. doi: 10.4183/aeb.2019.74, PMID: 31149063 PMC6535326

[248] Salazar-Viedma M Vergaño-Salazar JG Pastenes L D’Afonseca V . Simulation model for hashimoto autoimmune thyroiditis disease. Endocrinology. (2021) 162. doi: 10.1210/endocr/bqab190, PMID: 34496027 PMC8477452

[249] Ortega C Fernández S Estévez OA Aguado R Molina IJ Santamaría M . IL-17 producing T cells in celiac disease: angels or devils? Int Rev Immunol. (2013) 32:534–43. doi: 10.3109/08830185.2013.834898, PMID: 24040774

[250] Costa VS Mattana TCC da Silva MER . Unregulated IL-23/IL-17 immune response in autoimmune diseases. Diabetes Res Clin Pract. (2010) 88:222–6. doi: 10.1016/j.diabres.2010.03.014, PMID: 20392505

[251] Li H Tsokos GC . IL-23/IL-17 axis in inflammatory rheumatic diseases. Clin Rev Allergy Immunol. (2021) 60:31–45. doi: 10.1007/s12016-020-08823-4, PMID: 33185790 PMC8018566

[252] Hamamah S Barry F Vannier S Anahory T Haahtela T Antó JM . Infertility, IL-17, IL-33 and microbiome cross-talk: the extended ARIA-meDALL hypothesis. Int J Mol Sci. (2024) 25. doi: 10.3390/ijms252211981, PMID: 39596052 PMC11594021

[253] Ramos-Nino ME Obadiah AA Ozugha IO Ramdass PVAK . The association between asthma and endometriosis: A systematic review and metanalysis. J Respir. (2025) 5:6. doi: 10.3390/jor5020006, PMID: 41725453

[254] Doroudchi A Pathria M Modena BD . Asthma biologics. Ann Allergy Asthma Immunol. (2020) 124:44–56. doi: 10.1016/j.anai.2019.10.016, PMID: 31655122 PMC6911637

[255] Kelsen SG Agache IO Soong W Israel E Chupp GL Cheung DS . Astegolimab (anti-ST2) efficacy and safety in adults with severe asthma: A randomized clinical trial. J Allergy Clin Immunol. (2021) 148:790–8. doi: 10.1016/j.jaci.2021.03.044, PMID: 33872652

[256] Menzies-Gow A Corren J Bourdin A Chupp G Israel E Wechsler ME . Tezepelumab in adults and adolescents with severe, uncontrolled asthma. N Engl J Med. (2021) 384:1800–9. doi: 10.1056/NEJMoa2034975, PMID: 33979488

[257] Amisaki M Zebboudj A Yano H Zhang SL Payne G Chandra AK . IL-33-activated ILC2s induce tertiary lymphoid structures in pancreatic cancer. Nature. (2025) 638:1076–84. doi: 10.1038/s41586-024-08426-5, PMID: 39814891 PMC11864983

